# The Effect of Rural Versus Urban Residence on Risk of Unplanned Readmission and Death in Older Veterans Discharged From VA Hospitals

**DOI:** 10.1111/jrh.70183

**Published:** 2026-07-03

**Authors:** Joanne K. Daggy, Anthony J. Perkins, Katie Ross‐Driscoll, Laura J. Myers, Stanley E. Taylor, Mathew J. Reeves, Greg Arling, Indrakshi Roy, Scotte R. Hartronft, Cathy C. Schubert, Dawn M. Bravata

**Affiliations:** ^1^ Department of Biostatistics and Health Data Science Indiana University School of Medicine, Fairbanks School of Public Health Indianapolis Indiana USA; ^2^ Department of Veterans Affairs (VA) Health Systems Research (HSR) Expanding Expertise Through E‐health Network Development (EXTEND) Quality Enhancement Research Initiative (QUERI) Indianapolis Indiana USA; ^3^ Regenstrief Institute Indianapolis Indiana USA; ^4^ VA HSR Center for Health Information and Communication (CHIC) Richard L. Roudebush VA Medical Center Indianapolis Indiana USA; ^5^ Department of Medicine Indiana University School of Medicine Indianapolis Indiana USA; ^6^ Department of Epidemiology and Biostatistics Michigan State University East Lansing Michigan USA; ^7^ Department of Nursing Purdue University West Lafayette Indiana USA; ^8^ Department of Epidemiology and Biostatistics Indiana University School of Public Health Bloomington Indiana USA; ^9^ Office of Geriatrics & Extended Care Veterans Health Administration, U.S. Department of Veterans Affairs Washington, D.C. USA; ^10^ Community, Home, and Geriatrics Service, Richard L. Roudebush VA Medical Center Indianapolis Indiana USA

**Keywords:** disparity, geriatric, mortality, readmission, rural–urban

## Abstract

**Purpose:**

Evidence suggests that rural‐residing adults—compared with urban—may be at greater risk of readmission and mortality. Operational leaders within the Department of Veterans Affairs (VA) Geriatric Learning Health System (GLHS) sought to understand the effects of rural residence on readmission and mortality risk for older Veterans discharged from hospital to home.

**Methods:**

This observational cohort study included Veterans aged ≥ 65 years discharged from VA hospitals (fiscal year 2023) to home. A semi‐competing risk model was fit to jointly model unplanned readmission, mortality, and mortality after readmission, with rural residence as the exposure of interest. Data were censored at 30‐days, 90‐days, or 1‐year post discharge to examine short‐ and longer‐term effects. Additional variables considered for the model were identified through prior literature, clinical significance and those selected by the Centers for Medicare & Medicaid Services for unplanned readmission.

**Findings:**

Among 99,557 patients (120 hospitals), 28.4% were rural residents. Only 18% of rural residents lived within 30 min of a VA facility versus 80.3% of urban residents. At 30 days, compared to urban, rural‐residing patients had a 5% lower risk of readmission (hazard ratio [HR] = 0.95, 95% confidence interval [CI] = [0.91–1.00]), 20% higher risk of mortality (HR = 1.20, 95% CI = [1.03–1.40]), and a similarly higher risk of mortality after readmission (HR = 1.19, 95% CI = [1.03–1.39]).

**Conclusion:**

Rural‐residing older Veterans had lower risk of readmission but higher risk of mortality. These findings will guide future VA GLHSs: seeking modifiable factors (e.g., social drivers of health, timely services) associated with mortality risk among rural Veterans that can inform practice, policy, and quality improvement, thereby reducing disparities in outcomes.

## 1 Introduction

Unplanned hospital readmissions are a frequent and concerning outcome among older adults, with a substantial proportion experiencing readmission or death within one year of discharge [1, 2, 3]. For healthcare systems, unplanned 30‐day readmissions are a focus of national quality improvements programs such as the Hospital Readmissions Reduction Program [4], as they may signal deficiencies in care quality, particularly in transitional and post‐discharge support [5, 6, 7]. Rural residence has been associated with increased risk of 30‐day readmissions in older US adults [8] and in older Veterans [9]. Patients transitioning from hospital to home who live in rural areas may face greater hardships, compared with urban residents, when seeking timely healthcare given reduced availability of primary care and specialty care [7, 10, 11, 12, 13], fewer hospitals in rural areas [14], greater transportation barriers to receiving care [15], and reduced access to home healthcare and rehabilitation services [16]. In the Veteran population, rural residents have a higher prevalence of physical and mental health disability than urban residents [17], and access to healthcare is even more challenging for Veterans with multiple chronic service‐related conditions [18].

Prior studies have examined whether rural‐residing older patients, or specifically older Veterans, are at increased risk of an unplanned readmission but have focused only on specific cohorts [6, 19, 20] or consist of cohorts that are over 10 years old [8, 9, 11]. Using index admissions for older Veterans from 1997 to 2004 [9], rural‐residing older Veterans were found to have lower risk of unplanned readmission in 30 days among those discharged from a VA hospital (OR 0.97, 95% confidence interval [CI]: 0.96–0.98). In a broader population, using Medicare beneficiaries from 2000 to 2010 [11], no statistical difference was found in risk of readmission for those in rural settings versus urban. Among older US adults admitted for stroke, hip fracture, COPD, congestive heart failure, or pneumonia from 2011 to 2015, Kosar et al. [8] examined rural–urban differences in readmission (30‐, 90‐, 180‐day) and mortality and found those in rural counties had modestly higher adjusted rates of 30‐day readmission (9.7% vs. 9.2%) and mortality (1.7%:urban‐adjacent, 1.8%:urban‐nonadjacent vs. 1.4%:urban). Overall, a more contemporary study is needed to assess whether older rural‐residing Veterans discharged home differ in their risk of unplanned readmission.

The Department of Veterans Affairs (VA)—the largest integrated healthcare system in the country—is committed to implementing programs and policies that mitigate healthcare disparities for the 4.5 million rural‐dwelling Veterans [21]. The VA Geriatric Learning Health System (GLHS) was established to support community‐dwelling older Veterans as they transition from inpatient to outpatient settings [22]. The objective of this analysis was to determine whether disparities in readmissions exist for rural‐ versus urban‐residing Veterans transitioning from hospital to home that may inform future quality improvement or policy to improve transitional care.

## 2 Methods

### 2.1 Cohort Construction

This observational cohort included older Veterans (≥ 65 years) discharged from one of 120 VA medical or neurology wards for an unplanned admission in fiscal year 2023 and who were discharged to home in the community setting. Veterans discharged to other locations (e.g., nursing home) were excluded. Discharges from surgical/mental health were excluded as CMS does not include psychiatric admissions, and the needs of surgery patients are expected to be different than those of medicine/neurology. We used the VA Corporate Data Warehouse (CDW), which is a national repository of patient and clinical information [23], to identify Veterans who were discharged from a VA hospital in the one‐year study period. Veterans with hospice utilization in the one‐year prior to the index discharge and/or those with 29–30 days of hospice use following discharge were excluded.

### 2.2 Outcome Variables

Unplanned readmissions were identified according to the definition provided by the Centers for Medicare & Medicaid Services (CMS). Outcomes of interest were first unplanned readmission, mortality, and mortality after the first unplanned readmission. We considered events within 30‐days post discharge because this is the timeframe for the CMS HRRP program and is also clinically meaningful, as it captures the critical period during which hospitals and communities can collaborate to prevent avoidable readmissions [24]. To evaluate the short‐term and longer‐term effects of rural‐residency on outcomes, the time was censored at 30‐days, 90‐days, and 1‐year post discharge. Planned readmissions were not included in the outcome.

### 2.3 Explanatory Variables

The classification of residence as urban versus rural was based on Rural‐Urban Community Area (RUCA) codes that used the Veteran's geocoded zip code corresponding to their residential address [25]. The zip code in the quarter of the index hospital discharge was selected to determine urban versus rural status. If the zip code was not available during the quarter of discharge, the quarter following discharge or prior to discharge was used. The RUCA (census tract‐based) code ranges from 1 to 10 and considers population density as well as how closely a community is linked socio‐economically to larger urban centers. Urban areas are those with ≥ 5000 residents or ≥ 2000 housing units meeting population density and contiguity requirements [25]. Highly rural tracts have a RUCA score of 10 and are the remotest occupied land areas in which less than 10% of workers travel to urbanized areas or clusters. Rural areas are defined as all tracts not receiving scores in the urban or highly rural tiers. For our analysis, rural and highly rural settings were combined. Individuals living on the US insular island territories—Guam, American Samoa, Northern Mariana Islands, and the US Virgin Islands—are not coded with RUCA but receive a designation of “insular.” In our analysis, the nine Veterans designated as insular and 211 Veterans with unknown zip code were excluded (Supporting Information Figure S1).

All socio‐demographic characteristics were obtained from the VA CDW. Age at discharge, sex, race, marital status (married, widowed, other), Medicaid insurance, Medicare insurance, body mass index (BMI; calculated using height and weight measurements closest to the index date), and length of stay (number of midnights) of the index admission. Whether the patient lived alone was constructed using text‐mining of notes and/or health factor data. CMS risk variables include 32 variables that are used in risk adjustment of 30‐day unplanned readmission in CMS methodology [24]. Indicators for functional disability, cognitive impairment, and dementia were obtained using International Classification of Disease (ICD‐10) codes in the 1 year prior to the index admission. The Veterans Health Administration has developed a care assessment needs (CAN) score as a risk assessment tool for predicting mortality at 90 days, with higher scores indicating greater risk [26].

Patient‐level geographical variables include distance from the patient's residence to the nearest VA medical center (VAMC) or VA community‐based outpatient clinic (CBOC), which were further classified as drive time within 30 or 60 min. These variables were compared between rural‐ and urban‐residing patients but were not included in the statistical models since they are on the causal pathway, that is, travel distance/time to receive care may be a mediator through which being rural‐residing affects the risk of unplanned readmission and mortality [27].

Other available variables included: the readmission system‐of‐care (VA or community hospital) and the ICD‐10 codes for the index admission grouped into major diagnostic categories according to the Agency for Health Research and Quality (AHRQ) Clinical Classifications Software Refined (CCSR) Body System [28, 29]. Patients with evidence of hospice utilization prior to the index discharge were excluded. Although we sought to exclude anyone who received hospice, those with evidence of hospice or palliative care *consultations* were included in the primary analysis but were identified and excluded in a sensitivity analysis.

### 2.4 Statistical Methods

Patient characteristics were compared between rural‐ and urban‐residing groups with appropriate descriptive statistics such as mean and standard deviation or median and range for continuous variables, frequency and percents for categorical variables, and standardized differences [30]. Any socio‐demographic characteristic, CMS risk variable, or admission‐specific variable with a standardized difference greater than 5% was included in subsequent models as a potential confounder. Age was included a priori because it is known to be associated with increased risk of readmission and death. Although admission to an ICU is often used as a measure of patient disease severity, the focus of the Geriatric LHS is on transitional care outcomes post‐discharge. Therefore, discharged home from ICU was selected a priori as the most relevant to include in models.

As data were derived administratively, missing data were minimal. BMI had a negligible amount of missingness (0.078%); therefore, a separate category for missing BMI was created. The CAN score was missing for 1.5% of the cohort; because the CAN score is based on many of the other variables in the model (i.e., diagnosis codes and healthcare utilization), this score was not included in the modeling but was compared to ensure that scores were similar for rural and urban Veterans.

CMS methodology [24] for the assessment of 30‐day readmissions involves fitting a logistic regression model, but this approach ignores the impact of the competing event of death, as all patients are included in the denominator regardless of whether they survive to 30 days. We report the results of a logistic model for 30‐day readmissions using the CMS approach for comparison purposes only in the Supporting Information.

**FIGURE 1:**
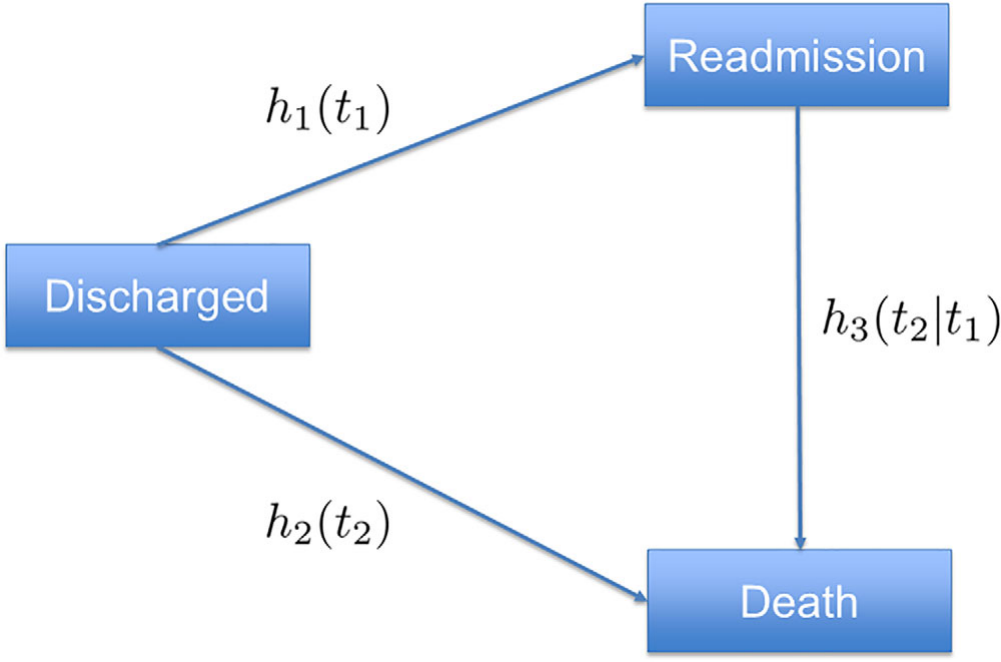
Semi‐competing risk model. Depiction of the multistate model, which is a semi‐competing risk model (illness‐death model) used to model the transition to readmission, death, or death after readmission.

Because our focus was on older patients, where mortality is higher, accounting for this competing event was necessary when seeking to understand differences in outcomes for rural‐ versus urban‐residing Veterans. Therefore, we employed a joint model to examine the readmission and mortality outcomes. Specifically, we employed a semi‐competing risk model with proportional hazards assumptions [31, 32] because readmission is a non‐terminal event, but death is a terminal event. Patients who did not experience a readmission or death within the specified time frame (30 days, 90 days, or 1 year) were censored at the end of follow‐up. Our primary interest as part of the Geriatric LHS was whether there was any difference in risk of readmission for rural‐residing versus urban with censoring at 30 days. We implemented the semi‐competing risk model to properly account for mortality. The risk for the longer time frames of 90 days and 1 year is considered exploratory. Therefore, we did not implement adjustment for multiple comparisons. The semi‐competing risk model, also known as the illness‐death model, is depicted in Figure 1. The semi‐competing risk model, assuming a Weibull proportional‐hazards model, was fit to risk of readmission, death, and death after readmission with censoring either at varying time frames such that the hazard for rural versus urban residence could be examined in the short‐term and longer time frames. We also assumed a less restrictive semi‐Markov assumption for the hazard of mortality after readmission, which means that the hazard of death after a readmission depends on the time since readmission. The semi‐competing risk model is characterized by three hazard functions that control the rate at which subjects transition between the states. The first hazard is for unplanned readmission, the second hazard for mortality, and the third hazard for mortality after readmission, where the baseline hazard is a function of time since readmission. In our model, we included covariates in each hazard.

$$h_{1}\left(t_{i1}|\gamma_{i},x_{i1}\right)=\gamma_{i}h_{01}\left(t_{i1}\right)\exp\left(x_{i1}^{T}\beta_{1}\right)\;\;\;\;t_{i1}>0$$

$$h_{2}\left(t_{i2}|\gamma_{i},x_{i2}\right)=\gamma_{i}h_{02}\left(t_{i2}\right)\text{exp}\left(x_{i2}^{T}\beta_{2}\right)\;\;\;\;t_{i2}>0$$

$$h_{3}\left(t_{i2}|t_{i1},\gamma_{i},x_{i3}\right)=\gamma_{i}h_{03}\left(z\left(t_{i1},t_{i2}\right)\right)\text{exp}\left(x_{i3}^{T}\beta_{3}\right)\;\;\;\;t_{i2}>t_{i1}$$

In this model, h_0g_ is an unspecified baseline hazard function, and β_g_ is a vector of log‐hazard ratio (HR) regression parameters associated with covariates x_ig_, and γ_i_ is a subject‐specific shared frailty term (i.e., random effect term) with a gamma(θ^−1^, θ^−1^) distribution with mean of 1 and variance θ; a γ_i_ between 0 and 1 indicates lower risk and γ_i_ >1 indicates higher risk. The shared frailty term is included to account for the dependency between unplanned readmission and mortality.

In Model 1, we fit the semi‐competing risk model with only rural versus urban residence included in each of the three hazards, and we fit the model separately with censoring at 30 days, 90 days, or 1 year. Model 2 is our primary model; it is fully adjusted for potential confounders and includes age, discharged home directly from the ICU, and any variable with a standardized difference > 5%. Model 3 includes all variables in Model 2 with the addition of readmission system‐of‐care (i.e., VA or community hospital) in the hazard for time to mortality after readmission.

For any model comparisons, likelihood ratio tests were performed by comparing the likelihood of the full versus the reduced model. Forest plots were generated to visually present the HRs and 95% CIs for the primary model. All analyses were conducted in SAS Enterprise v8.3 (SAS Institute, Cary, NC) and R 4.4.2. The semi‐competing risk models were fit using the R package SemiCompRisks [33].

### 2.5 Exploratory Analysis

Further analyses were conducted to examine how our results compare to prior literature [9]. Specifically, we examined whether the risk of mortality after readmission varied by the readmission system‐of‐care (i.e., whether the readmission occurred in a VA or community hospital) and whether the effect of rural‐residing on the risk of mortality after readmission varied by readmission system‐of‐care. This analysis was done by adding an indicator for readmission system‐of‐care and the two‐way interaction of readmission system‐of‐care and rural‐residing to the hazard of death after readmission in Model 2.

### 2.6 Sensitivity Analyses

Sensitivity analyses were conducted to confirm that our results were robust to our approach for analysis. As a sensitivity analysis, Cox frailty models were fit to readmission, mortality, and mortality after readmission separately incorporating stabilized weights. The stabilized weights are used to create a weighted sample where baseline covariates are balanced between urban and rural‐residing Veterans [34]. Weights were truncated at 99% and estimated via a logistic regression model including the same covariates as in our primary model to determine if the effect of rural residence on risk of readmission and mortality held if patients were equally likely to be rural‐ versus urban‐residing. As another sensitivity analysis, the primary model was estimated after removing Veterans with any evidence of hospice or palliative care *consultations* being placed.

### 2.7 Other Analyses

Because our initial model results indicated that rural‐residing Veterans have a higher risk of mortality, additional descriptive analysis included examining standardized differences in the proportions in each major diagnostic categories of the index admission between rural‐ and urban‐residing Veterans. Major diagnostic categories were coded using the ICD‐10‐CM Diagnoses User Guide, v2024.1 [29].

The study is reported following the Strengthening the Reporting of Observational Studies in Epidemiology (STROBE) reporting guideline [35]. The data that support the findings of this study must remain on Department of VA servers. Please contact the corresponding author if you are interested in working with these data.

### 2.8 Consent for Publication

This project was conducted as quality improvement and not research. Therefore, no human subjects research approval was sought.

## 3 Results

Our cohort consisted of 99,557 patients from 120 VA facilities with a mean age of 75.6 years (standard deviation: 7.0 years); 28.4% were rural‐residing, 22.6% were Black, 71.5% were White, and 5.9% were of other races. In our cohort, 82% were enrolled in Medicare, and 3.4% were enrolled in Medicaid. Most of the patients enrolled in Medicaid were also enrolled in Medicare: dual eligible (2714/3357, 80.9%). Of the 120 facilities, 13 (10.8%) were rural‐ and rural‐serving, 20 (16.7%) were urban‐ and rural‐serving, and 87 (72.5%) were urban‐ and not rural‐serving. At the patient level, the majority of Veterans (85,421/99,557, 85.8%) were discharged from an urban facility that was not rural‐serving.

Patient characteristics with standardized differences are reported in Table 1. Characteristics that differed by more than 5% between rural‐ and urban‐residing groups were: sex, race, marital status, living alone, BMI, insurance, admitted to an ICU, dementia, and the following eight CMS risk variables: endocrine and metabolic disorders, psychiatric comorbidity, cardiorespiratory failure and shock, coronary atherosclerosis or angina or cerebrovascular disease, arrhythmias or conduction disorders, chronic obstructive pulmonary disease, fibrosis of lung and other chronic lung disorders, and dialysis. Notably, no difference in the CAN 90‐day mortality score was observed between rural‐ and urban‐residing Veterans (standardized difference of −0.6%).

**TABLE 1:** Comparison of demographic and clinical characteristics by urban/rural status.

Characteristic	Rural/highly rural (*n* = 28,227)	Urban (*n* = 71,330)	Standardized difference (%)
**Socio‐demographic variables**			
Mean age (SD)	75.7 (6.6)	75.6 (7.2)	1.8
Male, *n* (%)	27,432 (97.2)	68,611 (96.2)	5.6
**Race, *n* (%)**			
Black	2429 (8.6)	20,039 (28.1)	−52.0
Other	1451 (5.1)	4465 (6.3)	−4.8
White	24,347 (86.3)	46,826 (65.6)	49.7
**Marital status, *n* (%)**			
Married	16,803 (59.5)	34,508 (48.4)	22.5
Other	8856 (31.4)	30,640 (43.0)	−24.1
Widowed	2568 (9.1)	6182 (8.7)	1.5
**Lives alone, *n* (%)**			
No	20,777 (73.6)	50,696 (71.1)	5.7
Yes	5289 (18.7)	15,813 (22.2)	−8.5
Unknown	2161 (7.7)	4821 (6.8)	3.5
**BMI**			
< 21	2547 (9.0)	8359 (11.7)	−8.8
21–24.9	5756 (20.4)	16,052 (22.5)	−5.1
25–29.9	9258 (32.8)	23,362 (32.8)	0.1
30–34.9	6292 (22.3)	14,059 (19.7)	6.3
35+	4348 (15.4)	9446 (13.2)	6.1
Missing	26 (0.1)	52 (0.1)	0.7
**Insurance**			
Medicaid	660 (2.3)	2697 (3.8)	−8.4
Medicare	23,386 (82.8)	58,212 (81.6)	3.2
**Admission specific variables**			
Median LOS (range)	4 (2, 60)	4 (2, 61)	0.2
Admitted to ICU, *n* (%)	3239 (11.5)	6995 (9.8)	5.4
ICU stay, *n* (%)	4349 (15.4)	9787 (13.7)	4.8
Discharged home directly from ICU, *n* (%)	1534 (5.4)	3256 (4.6)	4.0
**Other variables**			
CAN‐mortality score for 90 days, mean, (SD)^a^	84.7 (15.9)	84.8 (15.8)	−0.6
Dementia diagnosis (ICD‐10 year prior)	2148 (7.6)	6765 (9.5)	−6.7
Functional disability (ICD‐10 year prior)	4199 (14.9)	11,285 (15.8)	−2.6
Mild cognitive impairment (ICD‐10 year prior)	3761 (13.3)	9979 (14.0)	−1.9
**Geographical variables** ^c^			
Nearest VAMC—miles, mean (SD)	39.1 (26.6)	12.9 (13.9)	123.8
Median (range)	33.5 (0.0, 718.7)	8.7 (0, 258)	
Nearest CBOC—miles, mean (SD)^b^	27.6 (19.8)	13.7 (14.5)	80.2
Median (range)	24.3 (0.0 672.5)	8.9 (0.0, 169.0)	
Within 30‐min drive time of VAMC, *n* (%)	5088 (18.0)	57,252 (80.3)	−159.1
Within 60‐min drive time of VAMC, *n* (%)	17,280 (61.2)	68,852 (96.5)	−95.9
Within 30‐min drive time of CBOC, *n* (%)^b^	8593 (30.4)	54,661 (76.6)	−104.5
Within 60‐min drive time of CBOC, *n* (%)^b^	22,417 (79.4)	68,979 (96.7)	−55.3
**CMS risk variables, *n* (%)**			
Severe infection	343 (1.2)	870 (1.2)	0.0
Septicemia, sepsis, systemic inflammatory response syndrome/shock	2242 (7.9)	5463 (7.7)	1.1
Other infectious diseases and pneumonias	11,220 (39.8)	27,930 (39.2)	1.2
Metastatic cancer and acute leukemia	1399 (5.0)	3657 (5.1)	−0.8
Severe cancer	2961 (10.5)	7413 (10.4)	0.3
Other cancers	6442 (22.8)	15,974 (22.4)	1.0
Diabetes mellitus (DM) or DM complications	15,060 (53.4)	36,811 (51.6)	3.5
Protein‐calorie malnutrition	599 (2.1)	1925 (2.7)	−3.8
Other significant endocrine and metabolic disorders; disorders of fluid/electrolyte/acid‐base balance	23,156 (82.0)	55,843 (78.3)	9.4
End‐stage liver disease; cirrhosis of liver	1532 (5.4)	4220 (5.9)	−2.1
Pancreatic disease; peptic ulcer, hemorrhage, other specified gastrointestinal disorders	3871 (13.7)	9506 (13.3)	1.1
Rheumatoid arthritis and inflammatory connective tissue disease	1805 (6.4)	3866 (5.4)	4.1
Severe hematological disorders	307 (1.1)	762 (1.1)	0.2
Coagulation defects and other specified hematological disorders	2315 (8.2)	6085 (8.5)	−1.2
Iron deficiency or other/unspecified anemias and blood disease	10,612 (37.6)	27,235 (38.2)	−1.2
Drug/alcohol psychosis or dependence	6903 (24.5)	18,703 (26.2)	−4.1
Psychiatric comorbidity	10,955 (38.8)	30,321 (42.5)	−7.5
Hemiplegia, paraplegia, paralysis, functional disability	2289 (8.1)	5957 (8.4)	−0.9
Seizure disorders and convulsions	8117 (28.8)	19,003 (26.6)	4.7
Respirator dependence/tracheostomy status	225 (0.8)	493 (0.7)	1.2
Cardio‐respiratory failure and shock	4333 (15.4)	9344 (13.1)	6.4
Congestive heart failure	9494 (33.6)	22,722 (31.9)	3.8
Coronary atherosclerosis or angina, cerebrovascular disease	18,967 (67.2)	45,222 (63.4)	8.0
Specified arrhythmias and other heart rhythm disorders	12,792 (45.3)	28,701 (40.2)	10.3
Chronic obstructive pulmonary disease (COPD)	10,834 (38.4)	22,518 (31.6)	14.3
Fibrosis of lung or other chronic lung disorders	12,811 (45.4)	29,362 (41.2)	8.5
Transplants	372 (1.3)	976 (1.4)	−0.4
Dialysis status	591 (2.1)	2380 (3.3)	−7.7
Renal failure	10,146 (35.9)	26,301 (36.9)	−1.9
Decubitus ulcer or chronic skin ulcer	2357 (8.4)	5962 (8.4)	−0.1
Hip fracture/dislocation	798 (2.8)	1840 (2.6)	1.5

Standardized difference [30] between two groups for continuous and binary variables, respectively: $d=\frac{100(\bar{x_{1}}-\bar{x_{2}})}{\sqrt{\frac{s_{1}^{2}+s_{2}^{2}}{2}}}$ and $d=\frac{100(p_{1}-p_{2})}{\sqrt{\frac{p_{1}\left(1-p_{1}\right)+p_{2}\left(1-p_{2}\right)}{2}}}.$

^a^ The Clinical Assessment Needs (CAN) score was missing for 1.5% (437/28,227) of rural patients and 1.5% (1093/71,330) of urban patients.

^b^ CBOC refers to community‐based outpatient clinics operated by the VA.

^c^ Geographic variables are not considered in the model.

### 3.1 Risk of Adverse Outcomes Among Rural‐Residing Older Adults

**FIGURE 2:**
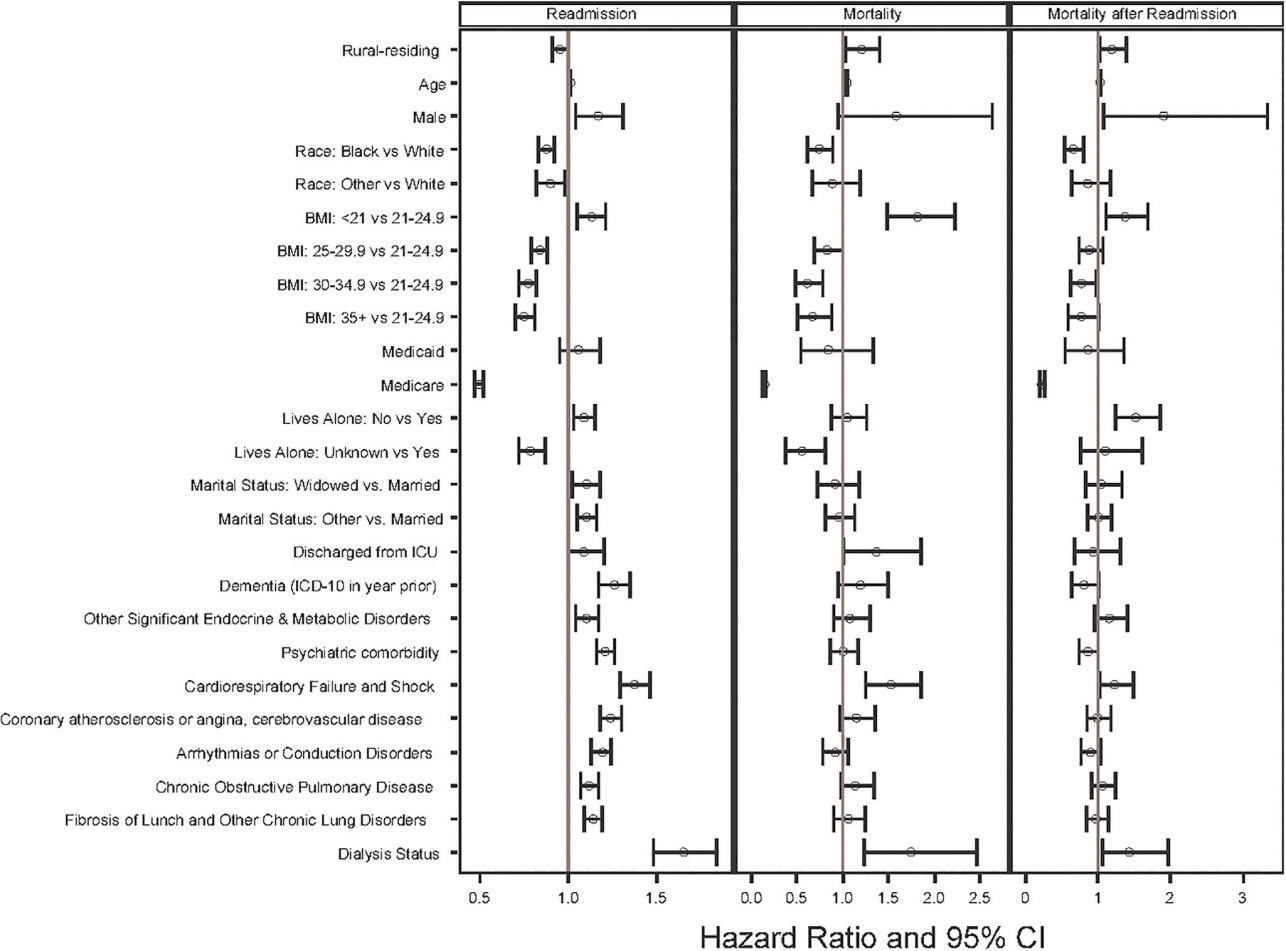
Hazard ratios (HRs) estimated from Model 2 for readmission, mortality, and mortality after readmission censored at 30 days. HRs estimated from semi‐competing risk model for readmission, mortality, and mortality after readmission censored at 30 days including the same variables for each hazard. *Note*: The HR for those missing BMI relative to 21–24.9 is not presented in the figure since out of range.

**FIGURE 3:**
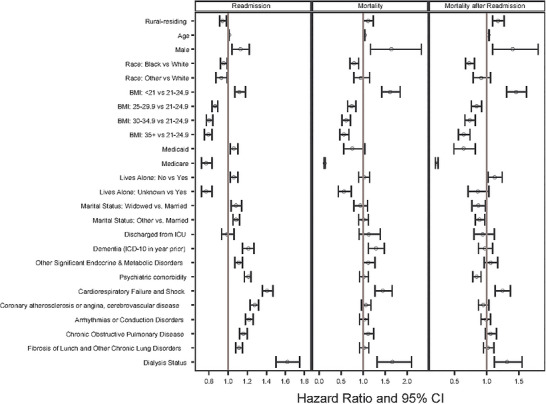
HRs estimated from Model 2 for readmission, mortality, and mortality after readmission censored at 90 days. HRs estimated from semi‐competing risk model for readmission, mortality, and mortality after readmission censored at 90 days including the same variables for each hazard. *Note*: The HR for those missing BMI relative to 21–24.9 is not presented in the figure since out of range.

**FIGURE 4:**
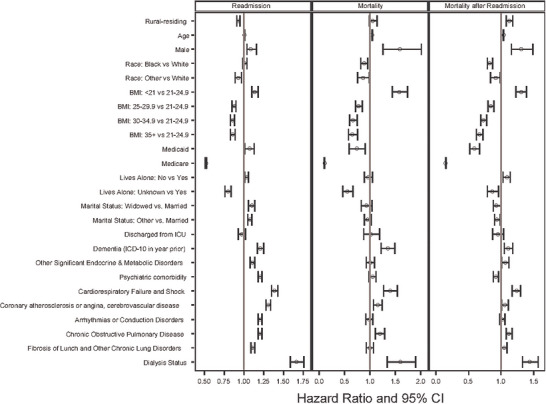
HRs estimated from Model 2 for readmission, mortality, and mortality after readmission censored at 1 year. HRs estimated from semi‐competing risk model for readmission, mortality, and mortality after readmission censored at 1 year including the same variables for each hazard. *Note*: The HR for those missing BMI relative to 21–24.9 is not presented in the figure since out of range.

The semi‐competing risk model evaluating time to readmission, time to mortality, and time to mortality after readmission, censored at 30 days, 90 days, or 1 year, with only the rural‐residing indicator included in each hazard (Model 1) indicates that rural‐residing Veterans were less likely to be readmitted and more likely to die (Supporting Information Table S4). Our primary model (Model 2), adjusted for characteristics that differed > 5% and age and discharge from ICU, had similar findings. The results of Model 2 are presented in Figures 2, 3, and 4 (Supporting Information Tables S1, S2, and S3). At 30 days, rural‐residing patients, compared with urban‐residing patients, were significantly less likely to be readmitted (HR 0.95 [95% CI, 0.91–0.999]) but had a 20% higher risk of mortality (HR 1.20 [95% CI, 1.03–1.40]), and a 19% higher risk of mortality after readmission (HR 1.19 [95% CI, 1.03–1.39]). At 90 days post discharge: rural‐residing patients still had a lower risk of readmission (HR 0.94 [95% CI, 0.91–0.98]; whereas, the increased risk of mortality was not statistically significant (HR 1.11 [95% CI, 1.00–1.23]), but the risk of mortality after readmission remained elevated (HR 1.18 [95% CI, 1.09–1.27]). At 1‐year post discharge: rural‐residing patients had a lower risk of readmission (HR 0.93 [95% CI, 0.91–0.95]), the increased risk of mortality was not statistically significant (HR 1.06 [95% CI, 0.99–1.14]), but the risk of death after readmission remained increased (HR 1.13 [95% CI, 1.08–1.18]).

Other patient characteristics that were associated with increased risk of unplanned readmission at 30 days from the multivariable model (Figure 2) were: older age; male sex; White race; low BMI; not enrolled in Medicare insurance; not living alone; being widowed or not being married; dementia; other significant endocrine and metabolic disorders; psychiatric comorbidity; cardio‐respiratory failure and shock; coronary atherosclerosis, angina, or cerebrovascular disease; arrhythmias or conduction disorders; chronic obstructive pulmonary disease; fibrosis of lung and other chronic lung disorders; and dialysis. Fewer variables were associated with death and death after readmission. (See Figure 2.)

### 3.2 Readmission System‐of‐Care

Rural‐residing Veterans sought care in the community (vs. the VA) more than urban‐residing patients: 26.9% (3906/14,532) of rural‐residing Veterans who were readmitted within 1 year of discharge were cared for in a community care hospital, compared to 17.5% (6733/38529) of urban‐residing Veterans. The mortality rate for those who were readmitted to a community care hospital was also higher than for those who were readmitted to a VA, at 30 days: mortality community care 8.1% versus VA 5.2%, at 90 days: mortality 16.6% versus VA 11.6%, and at 1 year: mortality community care 28.2% versus VA 22.9%.

Including the readmission system‐of‐care (at a community hospital vs. VA) in the hazard for time to mortality after readmission (Model 3) significantly improved the model fit, compared to Model 2 (Supporting Information Table S4, likelihood ratio test *p*‐values < 0.001). The HR for the risk of mortality after readmission for those readmitted to a community hospital relative to a VA hospital with censoring at 30‐days, 90‐days, and 1‐year, respectively were: HR = 1.72 (95% CI, 1.46–2.03), HR = 1.62 (95% CI, 1.49–1.76), and HR = 1.41 (95% CI, 1.35–1.48; available online only; Supporting Information Tables S5–S7). Thus, a Veteran with an unplanned readmission in 30 days that occurred at a community hospital had a 72% higher risk of mortality than a Veteran with an unplanned readmission in 30 days that occurred at a VA hospital. When an interaction term was added to each model between rural residence and readmission system‐of‐care, the interaction term was not statistically significant, indicating that the effect of rural residence on outcomes did not vary by readmission system‐of‐care.

**FIGURE 5:**
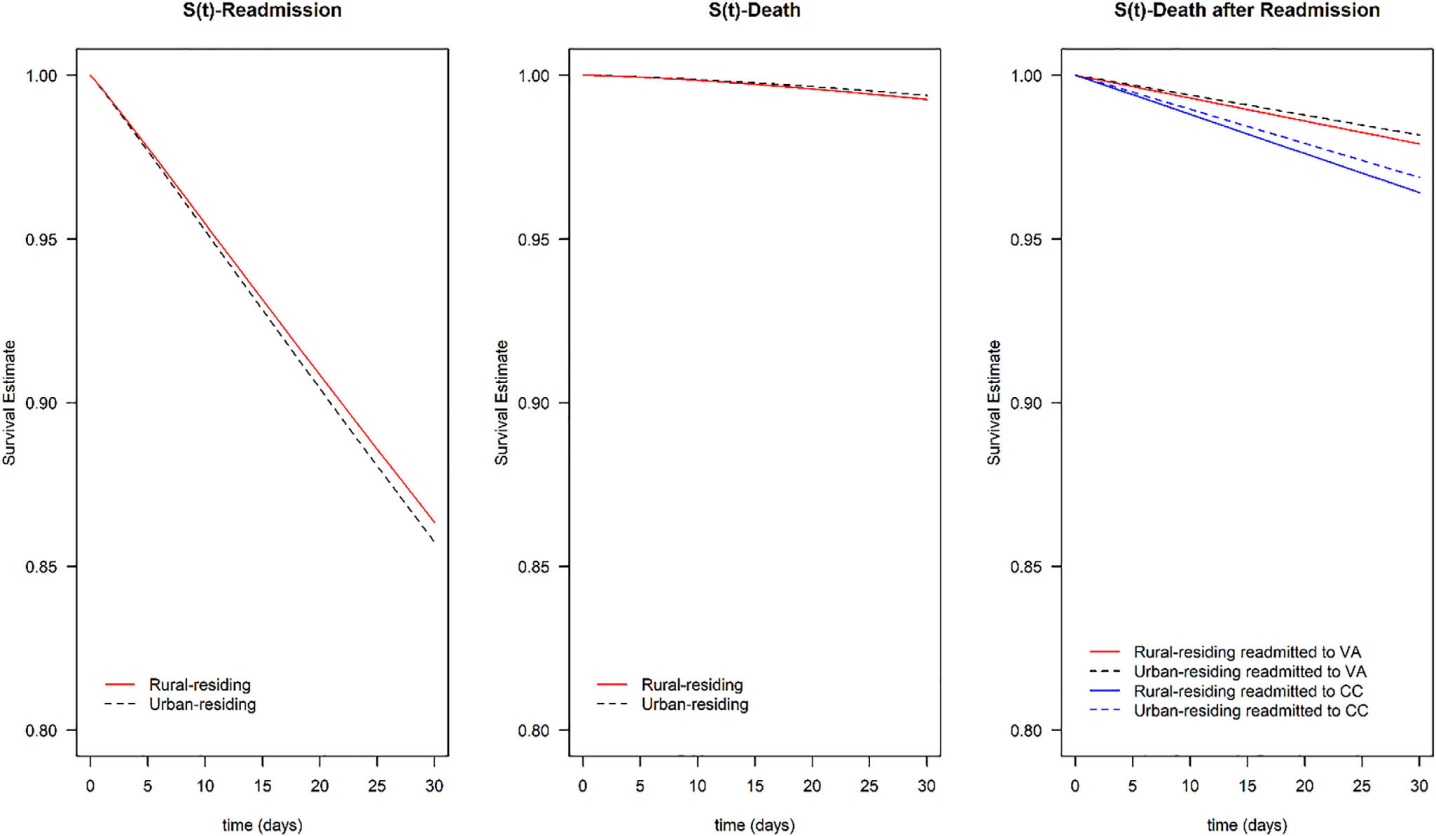
Survival estimates, $\hat{S}\left(t\right)$, for readmission, mortality, and mortality after readmission were obtained from Model 3, which includes the readmission system‐of‐care as a covariate in risk of mortality after readmission. Estimates are based on an average patient with covariates set as male, age 75 years, White, married, does not live alone, normal body mass index (BMI), no Medicaid, on Medicare, has other significant endocrine and metabolic disorder and coronary atherosclerosis or angina, and cerebrovascular disease.

Figure 5 provides the estimated survival curves obtained from Model 3 for an average patient for readmission, mortality, and mortality after readmission censored at 30 days by rural status and readmission system‐of‐care (for mortality after readmission). Based on Figure 5, there is a slightly higher mortality rate for rural‐residing patients (red solid line, middle panel) versus urban‐residing patients; and a slightly higher mortality rate after readmission for rural‐residing patients discharged from either a VA (red solid line, right panel) or community hospital (blue solid line, right panel), but regardless of rural status, patients discharged from a community hospital readmission had a much higher mortality rate than those discharged from a VA hospital readmission.

### 3.3 Sensitivity Analyses

Incorporating stabilized and truncated weights into Cox frailty models resulted in similar findings to the non‐weighted estimates (Supporting Information Tables S8–S10). Thus, if we consider a more causal interpretation through inverse‐probability weighting, our conclusions still hold in that rural‐residing patients had a decreased risk of readmission and increased risk of death.

After excluding the 9508 Veterans with any evidence of hospice *consultation* (Supporting Information Tables S11–S13), results were similar to the main findings, but the differences in rural‐ versus urban‐residing patients were more pronounced with HRs for the risk of mortality and risk of death after readmission farther from one. Specifically, the HR when censoring at 30 days for rural‐residing patients for readmission was HR 0.95 (95% CI, 0.90–1.00); *p* = 0.05, for risk of mortality was HR 1.25 (95% CI, 1.02–1.53), and mortality after readmission was HR 1.49 (95% CI, 1.15–1.93; Supporting Information Table S8).

### 3.4 Other Analyses

The proportion of patients discharged for each of the major diagnostic categories was examined for rural‐ and urban‐residing Veterans (Supporting Information Table S14). Rural‐residing patients had a slightly higher proportion being discharged for diseases of the respiratory system (standardized difference = 5.6%), but no other differences between rural‐ and urban‐residing Veterans were found (standardized differences were < 5% for all other major diagnosis categories).

## 4 Discussion

Our results indicate that rural‐residing older Veterans were less likely to be readmitted across all time frames than urban‐residing Veterans. Our study agrees with that of Weeks et al. [9], in that risk of unplanned readmission was lower for rural‐residing older Veterans who were discharged from a VA hospital. In contrast, Kosar et al. [8] reported that older rural‐residing adults discharged home were at increased risk for readmissions, though their analysis focused on select diagnoses and included patients from over a decade ago. In the intervening years, there has been widespread attention to inpatient care quality and attempts to reduce 30‐day readmissions. Future research is needed to examine readmission risk for rural‐residing patients using contemporary cohorts from non‐VA settings.

Our finding that rural residence was associated with increased risk of death is similar to the Kosar et al. [8] study (Supporting Information Table S16). Our study was not designed to examine why rural patients have higher mortality risk than urban patients, but observed differences in patient characteristics may provide some insights. For example, rural‐residing patients lived farther from a VA facility, and hence had longer drive times to receive healthcare services; over 80% of rural‐residing patients had to travel over 30 min to seek care at a VA facility. As found in prior research [36], these long drive times may be one reason that if readmitted, rural‐residing Veterans sought care at a community hospital (vs. the VA) more often than urban‐residing patients.

Interestingly, patients who were readmitted to a community hospital also had a higher mortality than those who were readmitted to a VA hospital. Lower rates of mortality at VA hospitals, compared with community hospitals, for Veteran patients has been observed previously [5]. This differential mortality between VA and community readmissions could be due to differential access to clinical information given that the VA electronic health record facilitates information sharing across all VA sites or to differences in care quality [5]. Future investigation is needed to understand whether community hospitals caring for rural‐dwelling Veterans consistently have worse outcomes, to identify patients who are at particularly high risk of post‐discharge death, and to examine patient characteristics that may be associated with post‐discharge death including social determinants of health.

The findings that rural‐residing patients had decreased risk of readmissions and increased risk of mortality were robust across analytic methods and sensitivity analyses. Using simple approaches such as logistic regression for the 30‐day readmission as well as the more statistically rigorous semi‐competing risk model resulted in the same conclusions. Additionally, the results held when using inverse‐probability weighting to obtain a more causal interpretation for the effect of rural‐residing on outcomes for this observational study.

Despite the methodological strengths of the study and the national scope of the cohort, limitations should be described. First, the cohort included older patients discharged from a VA hospital after a medical or neurological admission, which limits the generalizability of our findings to other healthcare systems, and to patients discharged from a mental health or surgical admission. Future cycles of the VA GLHS will seek to include index admissions from both VA and community hospitals to further examine differences in transitional care outcomes between rural‐ and urban‐residing patients. Second, most of our VA data are derived from medical records; future studies should consider detailed medical record review to improve our understanding of the causes of differences in risks of readmission and mortality between rural‐ and urban‐residing patients. Third, planned readmissions were not included in our model. As planned readmissions only occurred in 1.0% of our cohort within 30 days of discharge (vs. 15.7% unplanned), this limitation likely had minimal influence on results. Finally, the study did not account for any changes in residential address; however, a comparison of residential address to the quarter before and after index discharge showed that over 94% of addresses remained unchanged.

This study demonstrated that rural residence is associated with decreased risk of unplanned readmissions but increased risk of mortality among community‐dwelling older Veterans discharged from a hospital to home and that these risks persist across the one‐year post‐discharge period. The study also found that patients who were readmitted at community hospitals have increased risk of mortality, compared with patients who were readmitted at VA hospitals. Future research is needed to understand these urban–rural disparities to guide clinical decision‐making and healthcare policy, as some Veterans may choose whether to seek care at community or VA hospitals.

## Funding

This work was supported by the US Department of VA, Health Systems Research (HSR), Expanding Expertise Through E‐health Network Development Quality Enhancement Research Initiative (QUERI; QUE HX0003205‐01) The funding agency had no role in the design or conduct of the study; collection, management, analysis, or interpretation of the data; preparation, review, or approval of the manuscript; nor the decision to submit the manuscript for publication.

## Ethics Statement

The Geriatric LHS was a quality improvement project and was designated as not‐research. The comparison of risk of readmission for Veterans discharged home who were rural‐residing versus urban was part of the original proposal for the Geriatric LHS. Therefore, no approval from an Institutional Review Board was sought.

## Conflicts of Interest

The authors declare no conflicts of interest.

## Supporting information




**Supplemental Figure 1**: Cohort Information.
**Supplemental Figure 2**: Readmission: Hazard Ratios and 95% CI from Model 2 with censoring at 30 days, 90 days, and 1 year.
**Supplemental Figure 3**: Mortality: Hazard Ratios and 95% CI from Model 2 with censoring at 30 days, 90 days, and 1 year.
**Supplemental Figure 4**: Mortality after Readmission: Hazard Ratios and 95% CI from Model 2 with censoring at 30 days, 90 days, and 1 year.
**Supplemental Table 1 Model 2**: Semi‐competing risk model censored at 30 days.
**Supplemental Table 2 Model 2**: Semi‐competing risk model censored at 90 days.
**Supplemental Table 3 Model 2**: Semi‐competing risk model censored at 1 year.
**Supplemental Table 4**: Hazard Ratios and 95% CI for rural‐residing for Models 1 – 3.
**Supplemental Table 5 Model 3**: Semi‐competing risk model censored at 30 days with indicator of readmission system‐of‐care.
**Supplemental Table 6 Model 3**: Semi‐competing risk model censored at 90 days with indicator of readmission system‐of‐care.
**Supplemental Table 7 Model 3**: Semi‐competing risk model censored at 1 year with indicator of readmission system‐of‐care.
**Supplemental Table 8**: (Sensitivity analysis) Frailty models censored at 30 days for each outcome with IPTW using stabilized/truncated weights.
**Supplemental Table 9**: (Sensitivity analysis) Frailty models censored at 90 days for each outcome with IPTW using stabilized/truncated weights.
**Supplemental Table 10**: (Sensitivity analysis) Frailty models censored at 1 year for each outcome with IPTW using stabilized/truncated weights.
**Supplemental Table 11**: (Sensitivity analysis) Semi‐competing risk model censored at 30 days excluding hospice.
**Supplemental Table 12**: (Sensitivity analysis) Semi‐competing risk model censored at 90 days excluding hospice.
**Supplemental Table 13**: (Sensitivity analysis) Semi‐competing risk model censored at 1 year excluding hospice.
**Supplemental Table 14**: Major diagnostic categories for Index Admission.
**Supplemental Table 15**: Semi‐competing risk model censored at 30 days and logistic regression for readmission at 30 days for comparison only.
**Supplemental Table 16**: Frequencies and Percents for Outcomes.
