# Supplementary material for: The Effect of Rural Versus Urban Residence on Risk of Unplanned Readmission and Death in Older Veterans Discharged From VA Hospitals
Source: J Rural Health. 2026 Jul 3;42(3):e70183. doi: 10.1111/jrh.70183 (PMC13329746; doi:10.1111/jrh.70183)
Supplement: Supplementary file 1 — Supplemental Figure 1: Cohort Information. Supplemental Figure 2: Readmission: Hazard Ratios and 95% CI from Model 2 with censoring at 30 days, 90 days, and 1 year. Supplemental Figure 3: Mortality: Hazard Ratios and 95% CI from Model 2 with censoring at 30 days, 90 days, and 1 year. Supplemental Figure 4: Mortality after Readmission: Hazard Ratios and 95% CI from Model 2 with censoring at 30 days, 90 days, and 1 year. Supplemental Table 1 Model 2: Semi‐competing risk model censored at 30 days. Supplemental Table 2 Model 2: Semi‐competing risk model censored at 90 days. Supplemental Table 3 Model 2: Semi‐competing risk model censored at 1 year. Supplemental Table 4: Hazard Ratios and 95% CI for rural‐residing for Models 1 – 3. Supplemental Table 5 Model 3: Semi‐competing risk model censored at 30 days with indicator of readmission system‐of‐care. Supplemental Table 6 Model 3: Semi‐competing risk model censored at 90 days with indicator of readmission system‐of‐care. Supplemental Table 7 Model 3: Semi‐competing risk model censored at 1 year with indicator of readmission system‐of‐care. Supplemental Table 8: (Sensitivity analysis) Frailty models censored at 30 days for each outcome with IPTW using stabilized/truncated weights. Supplemental Table 9: (Sensitivity analysis) Frailty models censored at 90 days for each outcome with IPTW using stabilized/truncated weights. Supplemental Table 10: (Sensitivity analysis) Frailty models censored at 1 year for each outcome with IPTW using stabilized/truncated weights. Supplemental Table 11: (Sensitivity analysis) Semi‐competing risk model censored at 30 days excluding hospice. Supplemental Table 12: (Sensitivity analysis) Semi‐competing risk model censored at 90 days excluding hospice. Supplemental Table 13: (Sensitivity analysis) Semi‐competing risk model censored at 1 year excluding hospice. Supplemental Table 14: Major diagnostic categories for Index Admission. Supplemental Table 15: Semi‐competing risk model censored at 30 d [file JRH-42-0-s001.docx]

**Supporting Information (Online only)**

Supplemental Figure 1. Cohort Information

Supplemental Figure 2. Readmission: Hazard Ratios and 95% CI from Model 2 with censoring at 30 days, 90 days, and 1 year

Supplemental Figure 3. Mortality: Hazard Ratios and 95% CI from Model 2 with censoring at 30 days, 90 days, and 1 year

Supplemental Figure 4. Mortality after Readmission: Hazard Ratios and 95% CI from Model 2 with censoring at 30 days, 90 days, and 1 year

Supplemental Table 1. Model 2**:** Semi-competing risk model censored at 30 days

Supplemental Table 2. Model 2**:** Semi-competing risk model censored at 90 days

Supplemental Table 3. Model 2**:** Semi-competing risk model censored at 1 year

Supplemental Table 4. Hazard Ratios and 95% CI for rural-residing for Models 1 - 3

Supplemental Table 5. Model 3: Semi-competing risk model censored at 30 days with indicator of readmission system-of-care

Supplemental Table 6. Model 3: Semi-competing risk model censored at 90 days with indicator of readmission system-of-care

Supplemental Table 7. Model 3: Semi-competing risk model censored at 1 year with indicator of readmission system-of-care

Supplemental Table 8. (Sensitivity analysis) Frailty models censored at 30 days for each outcome with IPTW using stabilized/truncated weights

Supplemental Table 9. (Sensitivity analysis) Frailty models censored at 90 days for each outcome with IPTW using stabilized/truncated weights

Supplemental Table 10. (Sensitivity analysis) Frailty models censored at 1 year for each outcome with IPTW using stabilized/truncated weights

Supplemental Table 11. (Sensitivity analysis) Semi-competing risk model censored at 30 days excluding hospice

Supplemental Table 12. (Sensitivity analysis) Semi-competing risk model censored at 90 days excluding hospice

Supplemental Table 13. (Sensitivity analysis) Semi-competing risk model censored at 1 year excluding hospice

Supplemental Table 14. Major diagnostic categories for Index Admission

Supplemental Table 15. Semi-competing risk model censored at 30 days and logistic regression for readmission at 30 days for comparison only

Supplemental Table 16. Frequencies and Percents for Outcomes

**
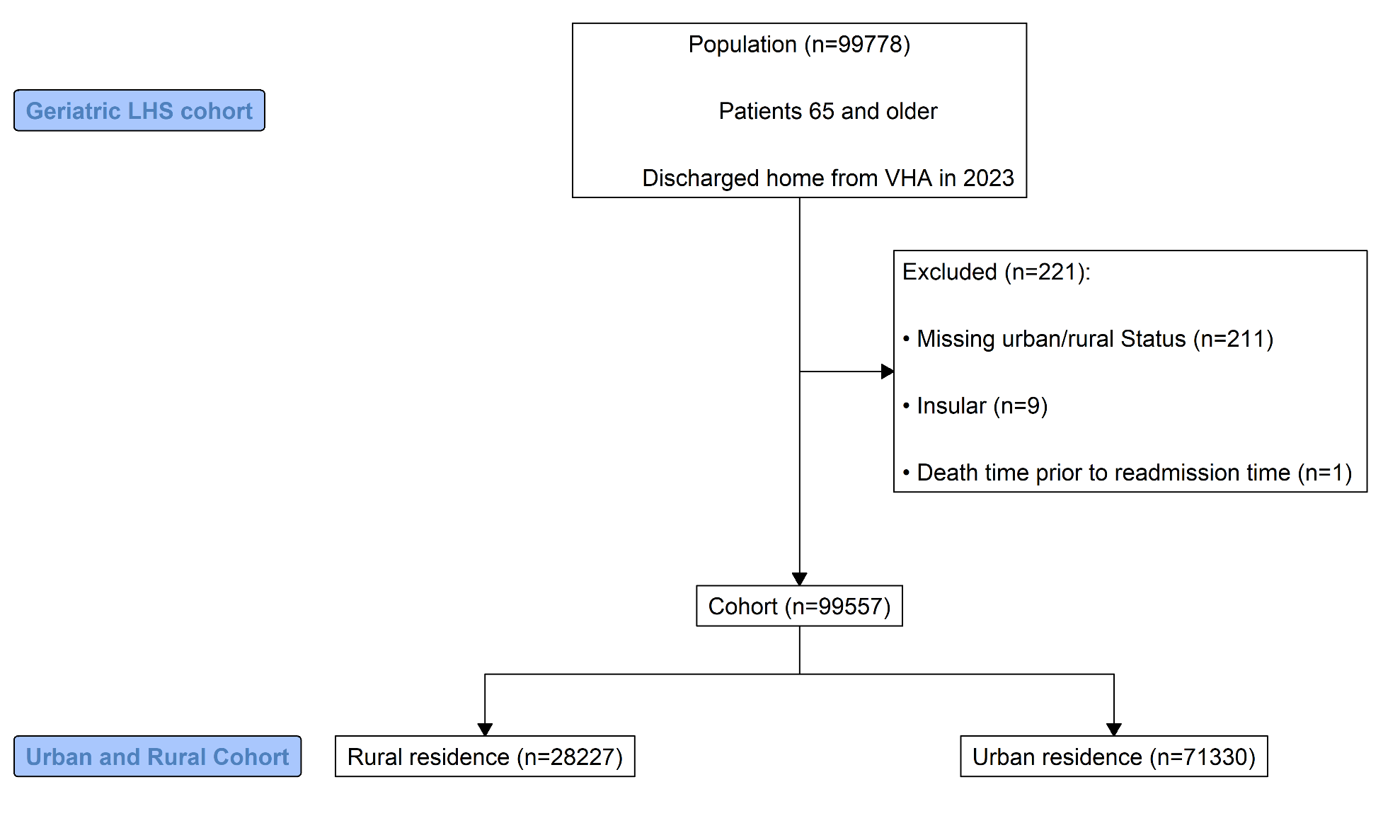
**

**Supplemental Figure 1:** Cohort Information

**
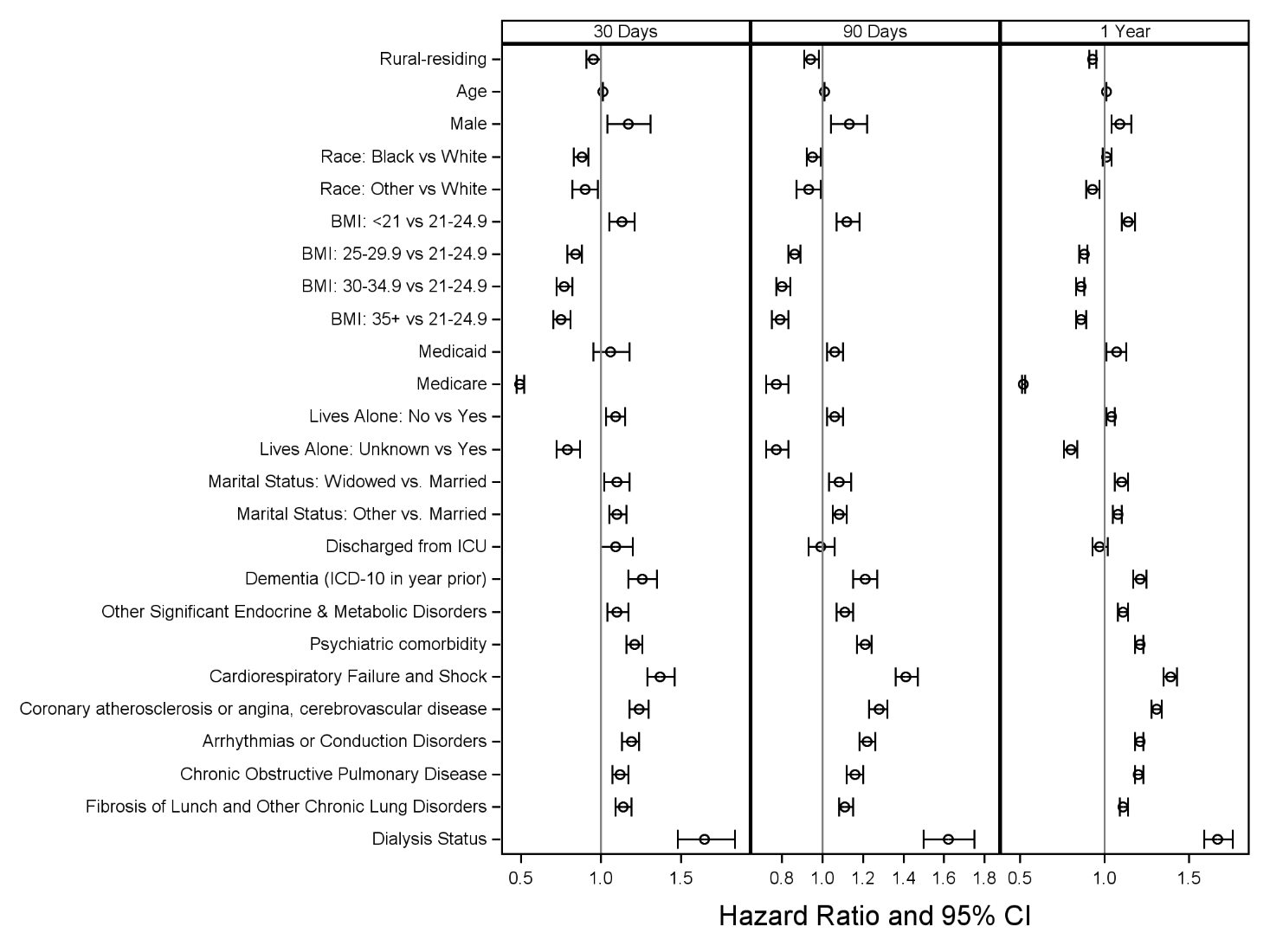
**

**Supplemental Figure 2.** Readmission: Hazard Ratios and 95% CI from Model 2 with censoring at 30 days, 90 days, and 1 year


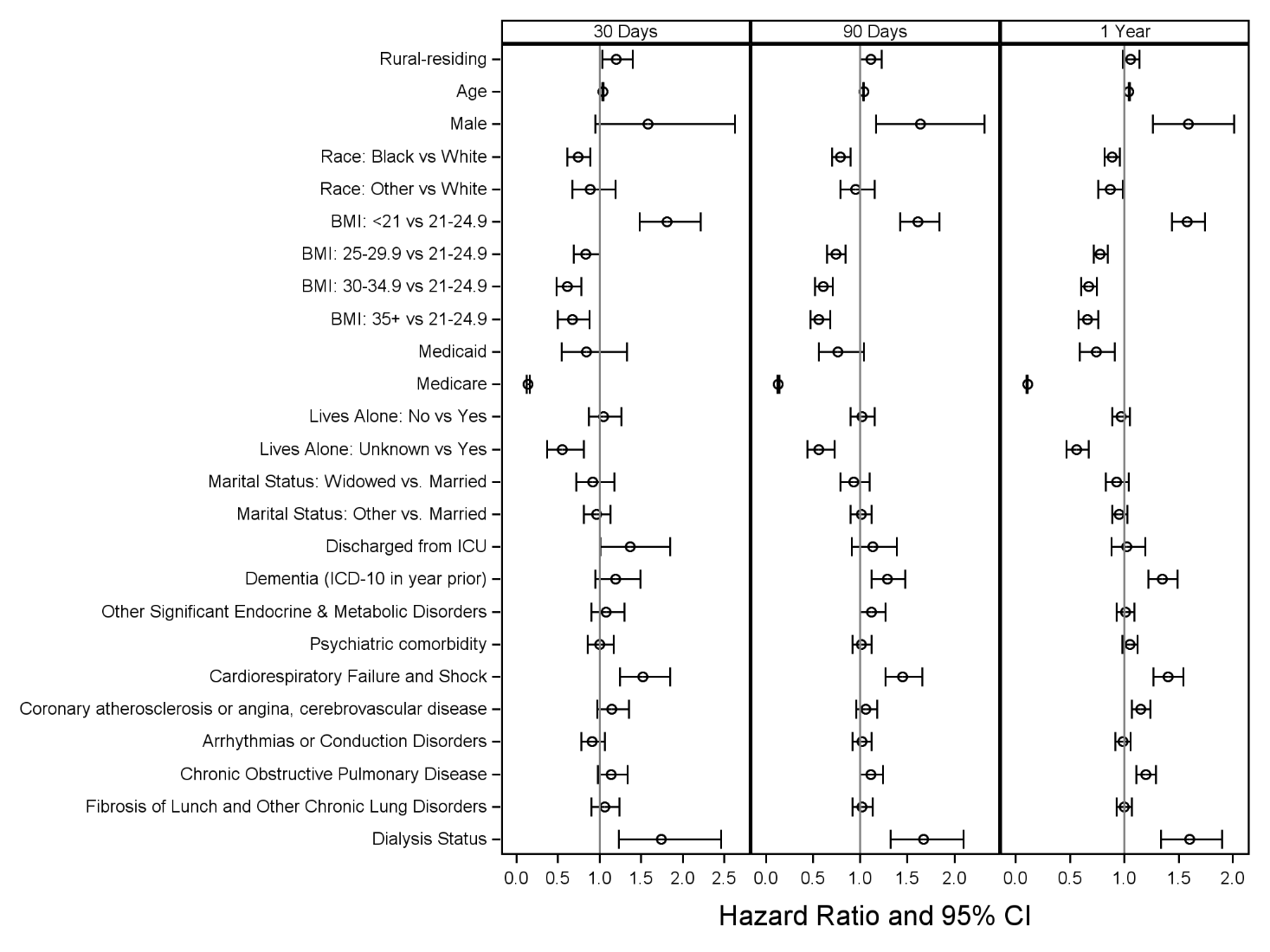


**Supplemental Figure 3.** Mortality: Hazard Ratios and 95% CI from Model 2 with censoring at 30 days, 90 days, and 1 year


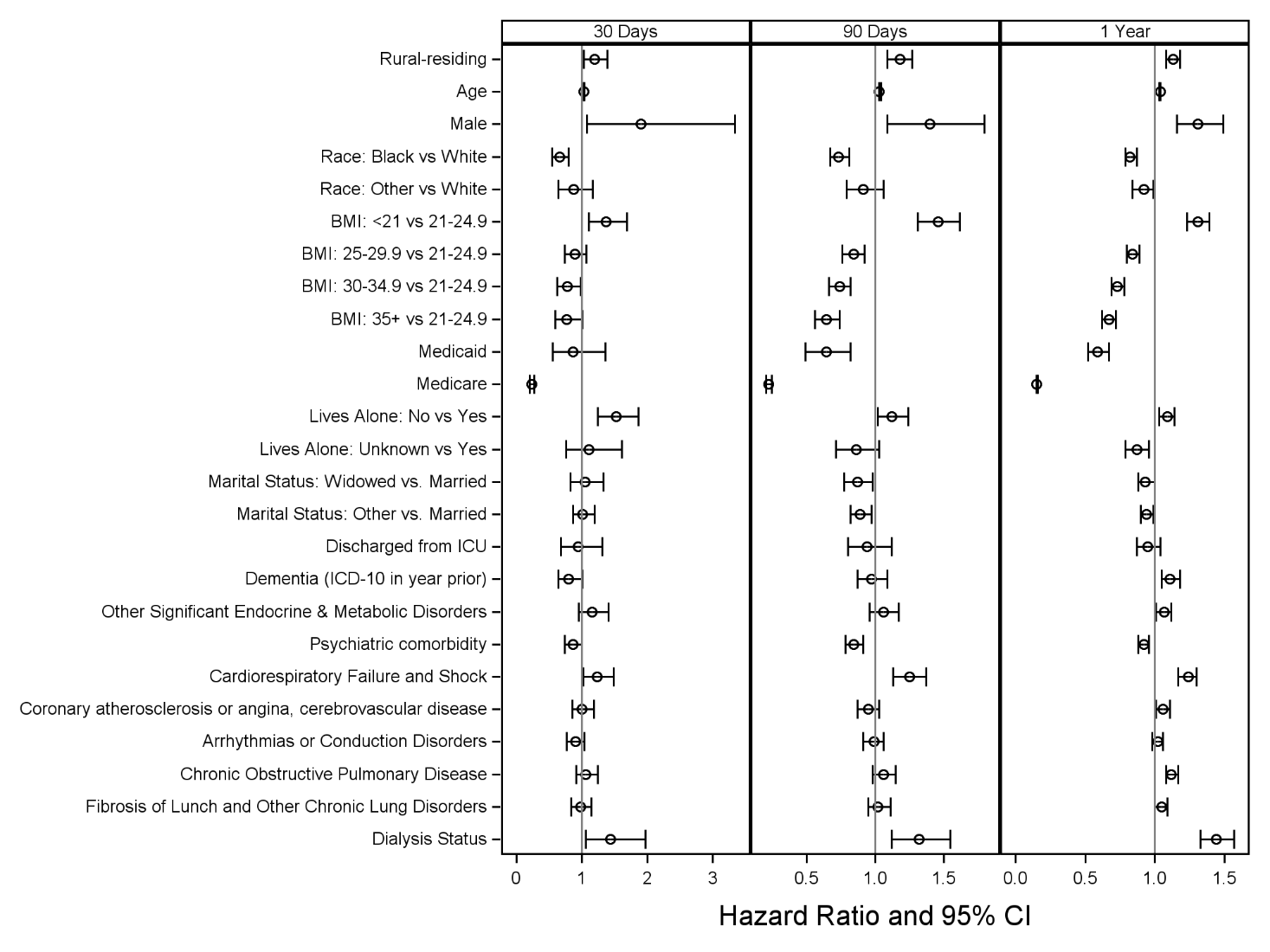


**Supplemental Figure 4:** Mortality after Readmission: Hazard Ratios and 95% CI from Model 2 with censoring at 30 days, 90 days, and 1 year

**Supplemental Table 1.** Model 2**:** Semi-competing risk model censored at 30 days

| **Characteristic** | **Readmission** | | **Mortality** | | **Mortality after Readmission** | |
| --- | --- | --- | --- | --- | --- | --- |
|  | **HR (95% CI)** | **P-value** | **HR (95% CI)** | **P-value** | **HR (95% CI)** | **P-value** |
| Rural-residing | 0.95 (0.91, 0.999) | 0.045 | 1.20 (1.03, 1.40) | 0.018 | 1.19 (1.03, 1.39) | 0.022 |
| Age ≥ 65 (years) | 1.01 (1.01, 1.01) | <0.001 | 1.04 (1.03, 1.05) | <0.001 | 1.03 (1.02, 1.04) | <0.001 |
| Male | 1.17 (1.04, 1.31) | 0.009 | 1.58 (0.95, 2.63) | 0.077 | 1.90 (1.08, 3.34) | 0.027 |
| **Race** |  |  |  |  |  |  |
| Black vs White | 0.88 (0.83, 0.92) | <0.001 | 0.74 (0.61, 0.89) | 0.001 | 0.66 (0.54, 0.80) | <0.001 |
| Other vs White | 0.90 (0.82, 0.98) | 0.016 | 0.89 (0.67, 1.19) | 0.444 | 0.87 (0.64, 1.17) | 0.340 |
| **BMI** |  |  |  |  |  |  |
| <21 vs 21-24.9 | 1.13 (1.05, 1.21) | 0.001 | 1.81 (1.48, 2.22) | <0.001 | 1.37 (1.11, 1.69) | 0.003 |
| 25-29.9 vs 21-24.9 | 0.84 (0.79, 0.88) | <0.001 | 0.83 (0.69, 1.00) | 0.053 | 0.89 (0.74, 1.07) | 0.225 |
| 30-34.9 vs 21-24.9 | 0.77 (0.72, 0.82) | <0.001 | 0.61 (0.48, 0.78) | <0.001 | 0.78 (0.62, 0.98) | 0.031 |
| 35+ vs 21-24.9 | 0.75 (0.70, 0.81) | <0.001 | 0.67 (0.50, 0.88) | 0.004 | 0.77 (0.59, 1.01) | 0.058 |
| Missing vs 21-24.9 | 0.52 (0.22, 1.25) | 0.144 | 2.69 (0.85, 8.50) | 0.093 | 2.25 (0.44, 11.56) | 0.333 |
| Medicaid | 1.06 (0.95, 1.18) | 0.330 | 0.84 (0.54, 1.33) | 0.467 | 0.86 (0.55, 1.36) | 0.526 |
| Medicare | 0.49 (0.47, 0.52) | <0.001 | 0.14 (0.12, 0.16) | <0.001 | 0.23 (0.20, 0.27) | <0.001 |
| **Lives Alone** |  |  |  |  |  |  |
| No vs Yes | 1.09 (1.03, 1.15) | 0.002 | 1.05 (0.87, 1.26) | 0.643 | 1.52 (1.24, 1.86) | <0.001 |
| Unknown vs Yes | 0.79 (0.72, 0.87) | <0.001 | 0.55 (0.37, 0.81) | 0.003 | 1.11 (0.76, 1.61) | 0.590 |
| **Marital Status** |  |  |  |  |  |  |
| Widowed vs. Married | 1.10 (1.02, 1.18) | 0.014 | 0.92 (0.72, 1.18) | 0.527 | 1.05 (0.83, 1.33) | 0.697 |
| Other vs. Married | 1.10 (1.05, 1.16) | <0.001 | 0.96 (0.81, 1.13) | 0.626 | 1.01 (0.86, 1.19) | 0.880 |
| Discharged from ICU | 1.09 (1.00, 1.20) | 0.063 | 1.37 (1.01, 1.85) | 0.041 | 0.94 (0.68, 1.31) | 0.708 |
| Dementia | 1.26 (1.17, 1.35) | <0.001 | 1.19 (0.95, 1.49) | 0.137 | 0.80 (0.64, 1.01) | 0.063 |
| Other Significant Endocrine & Metabolic Disorders | 1.10 (1.04, 1.17) | 0.001 | 1.08 (0.90, 1.30) | 0.387 | 1.16 (0.95, 1.41) | 0.145 |
| Psychiatric comorbidity | 1.21 (1.16, 1.26) | <0.001 | 1.00 (0.86, 1.17) | 0.959 | 0.86 (0.74, 1.00) | 0.052 |
| Cardiorespiratory Failure and Shock | 1.37 (1.29, 1.46) | <0.001 | 1.52 (1.25, 1.85) | <0.001 | 1.23 (1.02, 1.49) | 0.030 |

**Supplemental Table 1. (continued)**

| **Characteristic** | **Readmission** | | **Mortality** | | **Mortality after Readmission** | |
| --- | --- | --- | --- | --- | --- | --- |
|  | **HR (95% CI)** | **P-value** | **HR (95% CI)** | **P-value** | **HR (95% CI)** | **P-value** |
| Coronary atherosclerosis or angina, cerebrovascular disease | 1.24 (1.18, 1.30) | <0.001 | 1.15 (0.97, 1.35) | 0.100 | 1.00 (0.85, 1.18) | 0.991 |
| Arrhythmias or Conduction Disorders | 1.19 (1.13, 1.24) | <0.001 | 0.91 (0.78, 1.06) | 0.202 | 0.90 (0.77, 1.04) | 0.155 |
| Chronic Obstructive Pulmonary Disease | 1.12 (1.07, 1.17) | <0.001 | 1.14 (0.98, 1.34) | 0.093 | 1.06 (0.91, 1.24) | 0.443 |
| Fibrosis of Lunch and Other Chronic Lung Disorders | 1.14 (1.09, 1.19) | <0.001 | 1.06 (0.90, 1.24) | 0.472 | 0.98 (0.84, 1.15) | 0.800 |
| Dialysis Status | 1.65 (1.48, 1.84) | <0.001 | 1.74 (1.23, 2.46) | 0.002 | 1.44 (1.06, 1.97) | 0.021 |
| Other Model Information |  |  |  |  |  |  |
| Variance of frailty (95% CI) | 3.10 (2.69, 3.57) |  |  |  |  |  |
| Number of parameters | 85 |  |  |  |  |  |
| Log-likelihood | -107773.5 |  |  |  |  |  |
| LRT test (full model vs urban only), df = 75 | 46.8 | 0.996 |  |  |  |  |

Note: Wald test p-values reported for model parameters.

**Supplemental Table 2.** Model 2: Semi-competing risk model censored at 90 days

| **Characteristic** | **Readmission** | | **Mortality** | | **Mortality after Readmission** | |
| --- | --- | --- | --- | --- | --- | --- |
|  | **HR (95% CI)** | **P-value** | **HR (95% CI)** | **P-value** | **HR (95% CI)** | **P-value** |
| Rural-residing | 0.94 (0.91, 0.98) | 0.001 | 1.11 (1.00, 1.23) | 0.056 | 1.18 (1.09, 1.27) | <0.001 |
| Age ≥ 65 (years) | 1.01 (1.01, 1.01) | <0.001 | 1.04 (1.03, 1.04) | <0.001 | 1.03 (1.03, 1.04) | <0.001 |
| Male | 1.13 (1.04, 1.22) | 0.004 | 1.64 (1.17, 2.32) | 0.004 | 1.40 (1.09, 1.80) | 0.009 |
| **Race** |  |  |  |  |  |  |
| Black vs White | 0.95 (0.92, 0.99) | 0.007 | 0.79 (0.70, 0.90) | <0.001 | 0.73 (0.67, 0.81) | <0.001 |
| Other vs White | 0.93 (0.87, 0.99) | 0.017 | 0.95 (0.79, 1.15) | 0.622 | 0.91 (0.79, 1.06) | 0.234 |
| **BMI** |  |  |  |  |  |  |
| <21 vs 21-24.9 | 1.12 (1.07, 1.18) | <0.001 | 1.61 (1.42, 1.84) | <0.001 | 1.46 (1.31, 1.62) | <0.001 |
| 25-29.9 vs 21-24.9 | 0.86 (0.83, 0.89) | <0.001 | 0.74 (0.65, 0.84) | <0.001 | 0.84 (0.76, 0.92) | <0.001 |
| 30-34.9 vs 21-24.9 | 0.80 (0.77, 0.84) | <0.001 | 0.61 (0.52, 0.71) | <0.001 | 0.74 (0.66, 0.82) | <0.001 |
| 35+ vs 21-24.9 | 0.79 (0.75, 0.83) | <0.001 | 0.56 (0.47, 0.68) | <0.001 | 0.64 (0.56, 0.74) | <0.001 |
| Missing vs 21-24.9 | 0.34 (0.17, 0.69) | 0.003 | 1.90 (0.90, 4.02) | 0.095 | 1.23 (0.34, 4.37) | 0.754 |
| Medicaid | 1.06 (1.02, 1.10) | 0.003 | 0.76 (0.56, 1.04) | 0.085 | 0.64 (0.49, 0.82) | <0.001 |
| Medicare | 0.77 (0.72, 0.83) | <0.001 | 0.13 (0.12, 0.14) | <0.001 | 0.22 (0.20, 0.24) | <0.001 |
| **Lives Alone** |  |  |  |  |  |  |
| No vs Yes | 1.06 (1.02, 1.10) | 0.003 | 1.02 (0.90, 1.15) | 0.787 | 1.12 (1.02, 1.24) | 0.021 |
| Unknown vs Yes | 0.77 (0.72, 0.83) | <0.001 | 0.56 (0.44, 0.73) | <0.001 | 0.86 (0.71, 1.03) | 0.104 |
| **Marital Status** |  |  |  |  |  |  |
| Widowed vs. Married | 1.08 (1.03, 1.14) | 0.004 | 0.93 (0.79, 1.10) | 0.385 | 0.87 (0.77, 0.98) | 0.027 |
| Other vs. Married | 1.08 (1.05, 1.12) | <0.001 | 1.01 (0.90, 1.12) | 0.934 | 0.89 (0.82, 0.97) | 0.007 |
| Discharged from ICU | 0.99 (0.93, 1.06) | 0.814 | 1.13 (0.91, 1.39) | 0.272 | 0.94 (0.80, 1.12) | 0.499 |
| Dementia (ICD-10 in year prior) | 1.21 (1.15, 1.27) | <0.001 | 1.29 (1.12, 1.48) | 0.001 | 0.97 (0.87, 1.09) | 0.601 |
| Other Significant Endocrine & Metabolic Disorders | 1.11 (1.07, 1.15) | <0.001 | 1.12 (1.00, 1.27) | 0.057 | 1.06 (0.96, 1.17) | 0.255 |
| Psychiatric comorbidity | 1.21 (1.17, 1.24) | <0.001 | 1.01 (0.92, 1.12) | 0.835 | 0.84 (0.78, 0.91) | <0.001 |
| Cardiorespiratory Failure and Shock | 1.41 (1.36, 1.47) | <0.001 | 1.45 (1.27, 1.66) | <0.001 | 1.25 (1.13, 1.37) | <0.001 |
| Coronary atherosclerosis or angina, cerebrovascular disease | 1.28 (1.23, 1.32) | <0.001 | 1.06 (0.96, 1.18) | 0.262 | 0.95 (0.87, 1.03) | 0.229 |
| Arrhythmias or Conduction Disorders | 1.22 (1.18, 1.26) | <0.001 | 1.02 (0.92, 1.12) | 0.772 | 0.99 (0.91, 1.06) | 0.711 |

**Supplemental Table 2. (continued)**

| **Characteristic** | **Readmission** | | **Mortality** | | **Mortality after Readmission** | |
| --- | --- | --- | --- | --- | --- | --- |
|  | **HR (95% CI)** | **P-value** | **HR (95% CI)** | **P-value** | **HR (95% CI)** | **P-value** |
| Chronic Obstructive Pulmonary Disease | 1.16 (1.12, 1.20) | <0.001 | 1.11 (1.00, 1.24) | 0.046 | 1.06 (0.98, 1.15) | 0.120 |
| Fibrosis of Lunch and Other Chronic Lung Disorders | 1.11 (1.08, 1.15) | <0.001 | 1.02 (0.92, 1.13) | 0.743 | 1.02 (0.95, 1.11) | 0.583 |
| Dialysis Status | 1.62 (1.50, 1.75) | <0.001 | 1.67 (1.32, 2.10) | <0.001 | 1.32 (1.12, 1.55) | 0.001 |
| Other Model Information |  |  |  |  |  |  |
| Variance of frailty (95% CI) | 1.20 (1.08, 1.34) |  |  |  |  |  |
| Number of parameters | 85 |  |  |  |  |  |
| Log-likelihood | -226703 |  |  |  |  |  |
| LRT test (full model vs urban only), df = 75 | 117.7 | <0.001 |  |  |  |  |

Note: Wald test p-values reported for model parameters.

**Supplemental Table 3.** Model 2: Semi-competing risk model censored at 1 year

| **Characteristic** | **Readmission** | | **Mortality** | | **Mortality after Readmission** | |
| --- | --- | --- | --- | --- | --- | --- |
|  | **HR (95% CI)** | **P-value** | **HR (95% CI)** | **P-value** | **HR (95% CI)** | **P-value** |
| Rural-residing | 0.93 (0.91, 0.95) | <0.001 | 1.06 (0.99, 1.14) | 0.109 | 1.13 (1.08, 1.18) | <0.001 |
| Age | 1.01 (1.01, 1.01) | <0.001 | 1.04 (1.04, 1.05) | <0.001 | 1.04 (1.03, 1.04) | <0.001 |
| Male | 1.09 (1.04, 1.16) | 0.001 | 1.59 (1.26, 2.01) | <0.001 | 1.31 (1.16, 1.49) | <0.001 |
| **Race** |  |  |  |  |  |  |
| Black vs White | 1.01 (0.99, 1.04) | 0.297 | 0.89 (0.82, 0.96) | 0.004 | 0.82 (0.79, 0.87) | <0.001 |
| Other vs White | 0.93 (0.89, 0.97) | 0.001 | 0.87 (0.76, 0.99) | 0.034 | 0.92 (0.84, 0.99) | 0.032 |
| **BMI** |  |  |  |  |  |  |
| <21 vs 21-24.9 | 1.14 (1.10, 1.18) | <0.001 | 1.58 (1.44, 1.74) | <0.001 | 1.31 (1.23, 1.39) | <0.001 |
| 25-29.9 vs 21-24.9 | 0.88 (0.85, 0.90) | <0.001 | 0.78 (0.72, 0.85) | <0.001 | 0.84 (0.80, 0.89) | <0.001 |
| 30-34.9 vs 21-24.9 | 0.86 (0.83, 0.88) | <0.001 | 0.67 (0.60, 0.75) | <0.001 | 0.73 (0.69, 0.78) | <0.001 |
| 35+ vs 21-24.9 | 0.86 (0.83, 0.89) | <0.001 | 0.66 (0.58, 0.76) | <0.001 | 0.67 (0.62, 0.72) | <0.001 |
| Missing vs <21 | 0.29 (0.17, 0.49) | <0.001 | 1.40 (0.84, 2.34) | 0.203 | 0.79 (0.31, 2.04) | 0.630 |
| Medicaid | 1.07 (1.01, 1.13) | 0.016 | 0.74 (0.59, 0.91) | 0.005 | 0.59 (0.52, 0.67) | <0.001 |
| Medicare | 0.52 (0.51, 0.53) | <0.001 | 0.11 (0.10, 0.11) | <0.001 | 0.15 (0.15, 0.16) | <0.001 |
| **Lives Alone** |  |  |  |  |  |  |
| No vs Yes | 1.04 (1.01, 1.06) | 0.010 | 0.97 (0.89, 1.05) | 0.401 | 1.09 (1.03, 1.14) | 0.001 |
| Unknown vs Yes | 0.80 (0.76, 0.84) | <0.001 | 0.56 (0.47, 0.67) | <0.001 | 0.87 (0.79, 0.96) | 0.006 |
| **Marital Status** |  |  |  |  |  |  |
| Widowed vs. Married | 1.10 (1.06, 1.14) | <0.001 | 0.93 (0.83, 1.04) | 0.175 | 0.93 (0.88, 1.00) | 0.042 |
| Other vs. Married | 1.08 (1.05, 1.10) | <0.001 | 0.95 (0.89, 1.03) | 0.227 | 0.94 (0.90, 0.99) | 0.010 |
| Discharged from ICU | 0.97 (0.93, 1.02) | 0.179 | 1.02 (0.88, 1.19) | 0.805 | 0.95 (0.87, 1.04) | 0.283 |
| Dementia (ICD-10 in year prior) | 1.21 (1.17, 1.25) | <0.001 | 1.35 (1.22, 1.49) | <0.001 | 1.11 (1.05, 1.18) | <0.001 |
| Other Significant Endocrine & Metabolic Disorders | 1.11 (1.08, 1.14) | <0.001 | 1.01 (0.93, 1.09) | 0.828 | 1.07 (1.01, 1.12) | 0.019 |
| Psychiatric comorbidity | 1.21 (1.18, 1.23) | <0.001 | 1.05 (0.98, 1.12) | 0.210 | 0.92 (0.88, 0.96) | <0.001 |
| Cardiorespiratory Failure and Shock | 1.39 (1.35, 1.43) | <0.001 | 1.40 (1.27, 1.54) | <0.001 | 1.24 (1.17, 1.30) | <0.001 |
| Coronary atherosclerosis or angina, cerebrovascular disease | 1.31 (1.28, 1.34) | <0.001 | 1.15 (1.07, 1.24) | <0.001 | 1.06 (1.01, 1.11) | 0.015 |
| Arrhythmias or Conduction Disorders | 1.21 (1.18, 1.23) | <0.001 | 0.99 (0.92, 1.06) | 0.704 | 1.02 (0.98, 1.06) | 0.317 |

**Supplemental Table 3. (continued)**

| **Characteristic** | **Readmission** | | **Mortality** | | **Mortality after Readmission** | |
| --- | --- | --- | --- | --- | --- | --- |
|  | **HR (95% CI)** | **P-value** | **HR (95% CI)** | **P-value** | **HR (95% CI)** | **P-value** |
| Chronic Obstructive Pulmonary Disease | 1.20 (1.18, 1.23) | <0.001 | 1.20 (1.11, 1.29) | <0.001 | 1.12 (1.08, 1.17) | <0.001 |
| Fibrosis of Lunch and Other Chronic Lung Disorders | 1.11 (1.09, 1.14) | <0.001 | 1.00 (0.93, 1.07) | 0.933 | 1.05 (1.00, 1.09) | 0.034 |
| Dialysis Status | 1.67 (1.59, 1.76) | <0.001 | 1.60 (1.34, 1.90) | <0.001 | 1.44 (1.33, 1.57) | <0.001 |
| Other Model Information |  |  |  |  |  |  |
| Variance of frailty (95% CI) | 0.37 (0.33 0.41) |  |  |  |  |  |
| Number of parameters | 85 |  |  |  |  |  |
| Log-likelihood | -489019.3 |  |  |  |  |  |
| LRT test (full model vs urban only), df = 75 | 324.0 | <0.001 |  |  |  |  |

Note: Wald test p-values reported for model parameters.

**Supplemental Table 4:** Hazard Ratios and 95% CI for Rural vs Urban-residing Veterans for Models 1 – 3

| Model* | Log-likelihood | # of model  parameters | Time to readmission | Time to Mortality | Time to Mortality after Readmission |
| --- | --- | --- | --- | --- | --- |
|  |  |  | HR [95% CI] | HR [95% CI] | HR [95% CI] |
| **Model 1** |  |  |  |  |  |
| 30 day | -109529.2 | 10 | 0.970 [0.916, 1.028] | 1.227 [1.056, 1.427] | 1.280 [1.106, 1.482] |
| 90 day | -231,115.3 | 10 | 0.949 [0.913, 0.987] | 1.094 [0.989, 1.210] | 1.229 [1.139, 1.326] |
| 1 year | -501169.1 | 10 | 0.926 [0.901, 0.952] | 1.019 [0.950, 1.154] | 1.154 [1.106, 1.204] |
| **Model 2** |  |  |  |  |  |
| 30 day | -107773.5 | 85 | 0.954 [0.910, 0.990] | 1.202 [1.032, 1.400] | 1.194 [1.026, 1.390] |
| 90 day | -226703 | 85 | 0.944 [0.914, 0.975] | 1.105 [0.998, 1.225] | 1.177 [1.089, 1.272] |
| 1 year | -489019.3 | 85 | 0.929 [0.908, 0.950] | 1.061 [0.987, 1.140] | 1.131 [1.084, 1.180] |
| **Model 3** |  |  |  |  |  |
| 30 day | -107753.4 | 86 | 0.954 [0.910, 0.990] | 1.202 [1.032, 1.401] | 1.151 [0.988, 1.341] |
| 90 day | -226641.8 | 86 | 0.944 [0.914, 0.976] | 1.105 [0.997, 1.225] | 1.139 [1.053, 1.232] |
| 1 year | -488911.5 | 86 | 0.929 [0.908, 0.950] | 1.060 [0.987, 1.139] | 1.101 [1.055, 1.149] |

*Model 1 included rural residence only in each hazard. Model 2 was the fully adjusted model. Model 3 was the fully adjusted model that also included readmission system-of-care in the hazard for time to mortality after readmission.

**Supplemental Table 5.** Model 3: Semi-competing risk model censored at 30 days with indicator of readmission system-of-care

| **Characteristic** | **Readmission** | | **Mortality** | | **Mortality after Readmission** | |
| --- | --- | --- | --- | --- | --- | --- |
|  | **HR (95% CI)** | **P-value** | **HR (95% CI)** | **P-value** | **HR (95% CI)** | **P-value** |
| Rural-residing | 0.95 (0.91, 1.00) | 0.045 | 1.20 (1.03, 1.40) | 0.018 | 1.15 (0.99, 1.34) | 0.070 |
| Age ≥ 65 (years) | 1.01 (1.01, 1.01) | <0.001 | 1.04 (1.03, 1.05) | <0.001 | 1.03 (1.02, 1.04) | <0.001 |
| Male | 1.17 (1.04, 1.31) | 0.009 | 1.58 (0.95, 2.62) | 0.079 | 1.90 (1.08, 3.35) | 0.027 |
| **Race** |  |  |  |  |  |  |
| Black vs White | 0.88 (0.83, 0.92) | <0.001 | 0.74 (0.61, 0.89) | 0.001 | 0.66 (0.55, 0.81) | <0.001 |
| Other vs White | 0.90 (0.82, 0.98) | 0.015 | 0.89 (0.67, 1.19) | 0.442 | 0.85 (0.63, 1.14) | 0.277 |
| **BMI** |  |  |  |  |  |  |
| <21 vs 21-24.9 | 1.13 (1.05, 1.21) | 0.001 | 1.81 (1.48, 2.22) | <0.001 | 1.38 (1.12, 1.69) | 0.003 |
| 25-29.9 vs 21-24.9 | 0.84 (0.79, 0.88) | <0.001 | 0.83 (0.69, 1.00) | 0.052 | 0.89 (0.74, 1.07) | 0.227 |
| 30-34.9 vs 21-24.9 | 0.77 (0.72, 0.82) | <0.001 | 0.61 (0.48, 0.78) | <0.001 | 0.78 (0.62, 0.98) | 0.029 |
| 35+ vs 21-24.9 | 0.75 (0.70, 0.81) | <0.001 | 0.67 (0.50, 0.88) | 0.004 | 0.77 (0.59, 1.01) | 0.054 |
| Missing vs 21-24.9 | 0.53 (0.22, 1.26) | 0.152 | 2.74 (0.87, 8.64) | 0.085 | 2.48 (0.49, 12.65) | 0.274 |
| Medicaid | 1.06 (0.95, 1.18) | 0.329 | 0.84 (0.53, 1.33) | 0.466 | 0.85 (0.54, 1.33) | 0.468 |
| Medicare | 0.49 (0.47, 0.52) | <0.001 | 0.14 (0.12, 0.16) | <0.001 | 0.23 (0.20, 0.27) | <0.001 |
| **Lives Alone** |  |  |  |  |  |  |
| No vs Yes | 1.09 (1.03, 1.15) | 0.002 | 1.05 (0.87, 1.26) | 0.644 | 1.53 (1.25, 1.87) | <0.001 |
| Unknown vs Yes | 0.79 (0.72, 0.87) | <0.001 | 0.55 (0.37, 0.81) | 0.003 | 1.11 (0.76, 1.61) | 0.590 |
| **Marital Status** |  |  |  |  |  |  |
| Widowed vs. Married | 1.10 (1.02, 1.19) | 0.014 | 0.92 (0.72, 1.18) | 0.529 | 1.06 (0.84, 1.35) | 0.624 |
| Other vs. Married | 1.10 (1.05, 1.16) | <0.001 | 0.96 (0.81, 1.13) | 0.624 | 1.01 (0.86, 1.19) | 0.912 |
| Discharged from ICU | 1.09 (1.00, 1.20) | 0.063 | 1.37 (1.01, 1.85) | 0.042 | 0.95 (0.68, 1.32) | 0.736 |
| Dementia (ICD-10 in year prior) | 1.26 (1.17, 1.35) | <0.001 | 1.19 (0.95, 1.49) | 0.136 | 0.80 (0.63, 1.00) | 0.051 |
| Other Significant Endocrine & Metabolic Disorders | 1.10 (1.04, 1.17) | 0.001 | 1.08 (0.90, 1.30) | 0.386 | 1.14 (0.94, 1.38) | 0.195 |
| Psychiatric comorbidity | 1.21 (1.16, 1.26) | <0.001 | 1.00 (0.86, 1.17) | 0.961 | 0.86 (0.74, 1.00) | 0.044 |
| Cardiorespiratory Failure and Shock | 1.37 (1.29, 1.46) | <0.001 | 1.52 (1.25, 1.85) | <0.001 | 1.21 (1.00, 1.46) | 0.050 |
| Coronary atherosclerosis or angina, cerebrovascular disease | 1.24 (1.18, 1.30) | <0.001 | 1.15 (0.97, 1.35) | 0.100 | 0.98 (0.83, 1.16) | 0.804 |
| Arrhythmias or Conduction Disorders | 1.19 (1.13, 1.24) | <0.001 | 0.91 (0.78, 1.06) | 0.202 | 0.89 (0.76, 1.03) | 0.119 |

**Supplemental Table 5. (continued)**

| **Characteristic** | **Readmission** | | **Mortality** | | **Mortality after Readmission** | |
| --- | --- | --- | --- | --- | --- | --- |
|  | **HR (95% CI)** | **P-value** | **HR (95% CI)** | **P-value** | **HR (95% CI)** | **P-value** |
| Chronic Obstructive Pulmonary Disease | 1.12 (1.07, 1.17) | <0.001 | 1.14 (0.98, 1.34) | 0.094 | 1.05 (0.90, 1.22) | 0.576 |
| Fibrosis of Lunch and Other Chronic Lung Disorders | 1.14 (1.09, 1.19) | <0.001 | 1.06 (0.90, 1.24) | 0.470 | 0.98 (0.84, 1.14) | 0.781 |
| Dialysis Status | 1.65 (1.48, 1.85) | <0.001 | 1.74 (1.23, 2.46) | 0.002 | 1.46 (1.07, 2.00) | 0.017 |
| Readmission to Community Care |  |  |  |  | 1.72 (1.46, 2.03) | <0.001 |
| Other Model Information |  |  |  |  |  |  |
| Variance of frailty (95% CI) | 3.11 (2.70, 3.58) |  |  |  |  |  |
| Number of parameters | 86 |  |  |  |  |  |
| Log-likelihood | -107753.4 |  |  |  |  |  |
| LRT test (full model vs urban only), df = 76 | 46.7 | 0.997 |  |  |  |  |

Note: Wald test p-values reported for model parameters.

**Supplemental Table 6.** Model 3: Semi-competing risk model censored at 90 days with indicator of readmission system-of-care

| **Characteristic** | **Readmission** | | **Mortality** | | **Mortality after Readmission** | |
| --- | --- | --- | --- | --- | --- | --- |
|  | **HR (95% CI)** | **P-value** | **HR (95% CI)** | **P-value** | **HR (95% CI)** | **P-value** |
| Rural-residing | 0.94 (0.91, 0.98) | 0.001 | 1.11 (1.00, 1.23) | 0.056 | 1.14 (1.05, 1.23) | 0.001 |
| Age ≥ 65 (years) | 1.01 (1.01, 1.01) | <0.001 | 1.04 (1.03, 1.04) | <0.001 | 1.03 (1.03, 1.04) | <0.001 |
| Male | 1.13 (1.04, 1.22) | 0.003 | 1.65 (1.17, 2.32) | 0.004 | 1.40 (1.09, 1.80) | 0.009 |
| **Race** |  |  |  |  |  |  |
| Black vs White | 0.95 (0.92, 0.99) | 0.007 | 0.79 (0.70, 0.89) | <0.001 | 0.74 (0.68, 0.82) | <0.001 |
| Other vs White | 0.93 (0.87, 0.99) | 0.017 | 0.95 (0.79, 1.15) | 0.619 | 0.91 (0.78, 1.06) | 0.208 |
| **BMI** |  |  |  |  |  |  |
| <21 vs 21-24.9 | 1.12 (1.07, 1.18) | <0.001 | 1.62 (1.42, 1.84) | <0.001 | 1.46 (1.31, 1.62) | <0.001 |
| 25-29.9 vs 21-24.9 | 0.86 (0.83, 0.89) | <0.001 | 0.74 (0.65, 0.84) | <0.001 | 0.84 (0.76, 0.92) | <0.001 |
| 30-34.9 vs 21-24.9 | 0.80 (0.77, 0.84) | <0.001 | 0.61 (0.52, 0.71) | <0.001 | 0.73 (0.65, 0.82) | <0.001 |
| 35+ vs 21-24.9 | 0.79 (0.75, 0.83) | <0.001 | 0.56 (0.47, 0.68) | <0.001 | 0.64 (0.55, 0.73) | <0.001 |
| Missing vs 21-24.9 | 0.34 (0.17, 0.69) | 0.003 | 1.90 (0.89, 4.03) | 0.095 | 1.30 (0.37, 4.60) | 0.685 |
| Medicaid | 1.07 (0.99, 1.16) | 0.088 | 0.76 (0.56, 1.04) | 0.086 | 0.63 (0.49, 0.81) | <0.001 |
| Medicare | 0.49 (0.47, 0.51) | <0.001 | 0.13 (0.12, 0.14) | <0.001 | 0.22 (0.20, 0.24) | <0.001 |
| **Lives Alone** |  |  |  |  |  |  |
| No vs Yes | 1.06 (1.02, 1.10) | 0.003 | 0.93 (0.79, 1.10) | 0.388 | 1.13 (1.02, 1.24) | 0.017 |
| Unknown vs Yes | 0.77 (0.72, 0.83) | <0.001 | 0.93 (0.79, 1.10) | 0.388 | 0.87 (0.72, 1.04) | 0.128 |
| **Marital Status** |  |  |  |  |  |  |
| Widowed vs. Married | 1.08 (1.03, 1.14) | 0.004 | 0.93 (0.79, 1.10) | 0.388 | 0.88 (0.77, 0.99) | 0.036 |
| Other vs. Married | 1.08 (1.05, 1.12) | <0.001 | 1.01 (0.90, 1.12) | 0.933 | 0.89 (0.82, 0.97) | 0.006 |
| Discharged from ICU | 0.99 (0.93, 1.06) | 0.813 | 1.13 (0.91, 1.39) | 0.272 | 0.95 (0.80, 1.13) | 0.553 |
| Dementia (ICD-10 in year prior) | 1.21 (1.15, 1.27) | <0.001 | 1.29 (1.12, 1.48) | 0.001 | 0.96 (0.86, 1.08) | 0.506 |
| Other Significant Endocrine & Metabolic Disorders | 1.11 (1.07, 1.15) | <0.001 | 1.12 (1.00, 1.27) | 0.057 | 1.05 (0.95, 1.15) | 0.368 |
| Psychiatric comorbidity | 1.21 (1.17, 1.25) | <0.001 | 1.01 (0.92, 1.12) | 0.830 | 0.84 (0.78, 0.90) | <0.001 |
| Cardiorespiratory Failure and Shock | 1.42 (1.36, 1.48) | <0.001 | 1.45 (1.27, 1.66) | <0.001 | 1.23 (1.12, 1.36) | <0.001 |
| Coronary atherosclerosis or angina, cerebrovascular disease | 1.28 (1.24, 1.32) | <0.001 | 1.06 (0.96, 1.18) | 0.261 | 0.94 (0.86, 1.02) | 0.118 |
| Arrhythmias or Conduction Disorders | 1.22 (1.18, 1.26) | <0.001 | 1.02 (0.92, 1.12) | 0.766 | 0.98 (0.91, 1.06) | 0.558 |

**Supplemental Table 6. (continued)**

| **Characteristic** | **Readmission** | | **Mortality** | | **Mortality after Readmission** | |
| --- | --- | --- | --- | --- | --- | --- |
|  | **HR (95% CI)** | **P-value** | **HR (95% CI)** | **P-value** | **HR (95% CI)** | **P-value** |
| Chronic Obstructive Pulmonary Disease | 1.16 (1.12, 1.20) | <0.001 | 1.11 (1.00, 1.24) | 0.045 | 1.05 (0.97, 1.14) | 0.205 |
| Fibrosis of Lunch and Other Chronic Lung Disorders | 1.11 (1.08, 1.15) | <0.001 | 1.02 (0.92, 1.13) | 0.741 | 1.02 (0.94, 1.10) | 0.613 |
| Dialysis Status | 1.62 (1.50, 1.75) | <0.001 | 1.67 (1.32, 2.11) | <0.001 | 1.31 (1.12, 1.54) | 0.001 |
| Readmission to Community Care |  |  |  |  | 1.62 (1.49, 1.76) | <0.001 |
| Other Model Information |  |  |  |  |  |  |
| Variance of frailty (95% CI) | 1.21 (1.09, 1.35) |  |  |  |  |  |
| Number of parameters | 86 |  |  |  |  |  |
| Log-likelihood | -226641.8 |  |  |  |  |  |
| LRT test (full model vs urban only), df = 75 | 117.7 | 0.001 |  |  |  |  |

Note: Wald test p-values reported for model parameters.

**Supplemental Table 7.** Model 3: Semi-competing risk model censored at 1 year with indicator of readmission system-of-care

| **Characteristic** | **Readmission** | | **Mortality** | | **Mortality after Readmission** | |
| --- | --- | --- | --- | --- | --- | --- |
|  | **HR (95% CI)** | **P-value** | **HR (95% CI)** | **P-value** | **HR (95% CI)** | **P-value** |
| Rural-residing | 0.93 (0.91, 0.95) | <0.001 | 1.06 (0.99, 1.14) | 0.111 | 1.10 (1.06, 1.15) | <0.001 |
| Age | 1.01 (1.01, 1.01) | <0.001 | 1.04 (1.04, 1.05) | <0.001 | 1.04 (1.04, 1.04) | <0.001 |
| Male | 1.09 (1.04, 1.16) | 0.001 | 1.59 (1.26, 2.01) | <0.001 | 1.32 (1.16, 1.50) | <0.001 |
| **Race** |  |  |  |  |  |  |
| Black vs White | 1.01 (0.99, 1.04) | 0.306 | 0.89 (0.82, 0.96) | 0.004 | 0.84 (0.80, 0.88) | <0.001 |
| Other vs White | 0.93 (0.89, 0.97) | 0.001 | 0.87 (0.76, 0.99) | 0.033 | 0.92 (0.85, 0.99) | 0.035 |
| **BMI** |  |  |  |  |  |  |
| <21 vs 21-24.9 | 1.14 (1.10, 1.18) | <0.001 | 1.58 (1.44, 1.74) | <0.001 | 1.31 (1.23, 1.39) | <0.001 |
| 25-29.9 vs 21-24.9 | 0.88 (0.85, 0.90) | <0.001 | 0.78 (0.71, 0.85) | <0.001 | 0.84 (0.80, 0.88) | <0.001 |
| 30-34.9 vs 21-24.9 | 0.85 (0.83, 0.88) | <0.001 | 0.67 (0.60, 0.75) | <0.001 | 0.73 (0.68, 0.77) | <0.001 |
| 35+ vs 21-24.9 | 0.85 (0.83, 0.89) | <0.001 | 0.66 (0.58, 0.75) | <0.001 | 0.66 (0.61, 0.71) | <0.001 |
| Missing vs <21 | 0.29 (0.17, 0.49) | <0.001 | 1.40 (0.83, 2.34) | 0.204 | 0.80 (0.31, 2.06) | 0.644 |
| Medicaid | 1.07 (1.01, 1.13) | 0.016 | 0.74 (0.59, 0.91) | 0.005 | 0.59 (0.51, 0.67) | <0.001 |
| Medicare | 0.52 (0.50, 0.53) | <0.001 | 0.11 (0.10, 0.11) | <0.001 | 0.15 (0.15, 0.16) | <0.001 |
| **Lives Alone** |  |  |  |  |  |  |
| No vs Yes | 1.04 (1.01, 1.06) | 0.010 | 0.97 (0.89, 1.05) | 0.404 | 1.09 (1.04, 1.15) | 0.001 |
| Unknown vs Yes | 0.80 (0.76, 0.84) | <0.001 | 0.56 (0.47, 0.66) | <0.001 | 0.87 (0.79, 0.96) | 0.006 |
| **Marital Status** |  |  |  |  |  |  |
| Widowed vs. Married | 1.10 (1.06, 1.14) | <0.001 | 0.93 (0.83, 1.04) | 0.181 | 0.94 (0.88, 1.00) | 0.062 |
| Other vs. Married | 1.08 (1.05, 1.10) | <0.001 | 0.96 (0.89, 1.03) | 0.233 | 0.94 (0.90, 0.98) | 0.008 |
| Discharged from ICU | 0.97 (0.93, 1.01) | 0.175 | 1.03 (0.88, 1.19) | 0.754 | 0.96 (0.87, 1.05) | 0.338 |
| Dementia (ICD-10 in year prior) | 1.21 (1.17, 1.25) | <0.001 | 1.35 (1.22, 1.49) | <0.001 | 1.11 (1.04, 1.17) | 0.001 |
| Other Significant Endocrine & Metabolic Disorders | 1.11 (1.08, 1.14) | <0.001 | 1.01 (0.93, 1.09) | 0.823 | 1.06 (1.00, 1.11) | 0.040 |
| Psychiatric comorbidity | 1.21 (1.18, 1.23) | <0.001 | 1.05 (0.98, 1.12) | 0.203 | 0.92 (0.88, 0.96) | <0.001 |
| Cardiorespiratory Failure and Shock | 1.39 (1.35, 1.44) | <0.001 | 1.40 (1.28, 1.54) | <0.001 | 1.23 (1.17, 1.29) | <0.001 |
| Coronary atherosclerosis or angina, cerebrovascular disease | 1.31 (1.28, 1.34) | <0.001 | 1.15 (1.07, 1.24) | <0.001 | 1.05 (1.00, 1.10) | 0.040 |
| Arrhythmias or Conduction Disorders | 1.21 (1.18, 1.23) | <0.001 | 0.99 (0.92, 1.06) | 0.719 | 1.02 (0.98, 1.06) | 0.379 |

**Supplemental Table 7.** Semi-competing risk model censored at 1 year with indicator of readmission system-of-care

| **Characteristic** | **Readmission** | | **Mortality** | | **Mortality after Readmission** | |
| --- | --- | --- | --- | --- | --- | --- |
|  | **HR (95% CI)** | **P-value** | **HR (95% CI)** | **P-value** | **HR (95% CI)** | **P-value** |
| Chronic Obstructive Pulmonary Disease | 1.20 (1.18, 1.23) | <0.001 | 1.20 (1.11, 1.29) | <0.001 | 1.12 (1.07, 1.16) | <0.001 |
| Fibrosis of Lunch and Other Chronic Lung Disorders | 1.11 (1.09, 1.14) | <0.001 | 1.00 (0.93, 1.07) | 0.938 | 1.05 (1.01, 1.09) | 0.028 |
| Dialysis Status | 1.68 (1.59, 1.76) | <0.001 | 1.60 (1.35, 1.90) | <0.001 | 1.44 (1.32, 1.57) | <0.001 |
| Readmission to Community Care |  |  |  |  | 1.41 (1.35, 1.48) | <0.001 |
| Other Model Information |  |  |  |  |  |  |
| Variance of frailty (95% CI) | 0.38 (0.34 0.42) |  |  |  |  |  |
| Number of parameters | 86 |  |  |  |  |  |
| Log-likelihood | -488911.5 |  |  |  |  |  |
| LRT test (full model vs urban only), df = 75 | 322.6 | <0.001 |  |  |  |  |

Note: Wald test p-values reported for model parameters.

**Supplemental Table 8.** Individual frailty models for each outcome censored at 30 days with Inverse-Probability of Treatment Weights (IPTW) using stabilized and truncated weights (99^th^ percentile)

| **Characteristic** | **Readmission** | | **Mortality** | | **Mortality after Readmission** | |
| --- | --- | --- | --- | --- | --- | --- |
|  | **HR (95% CI)** | **P-value** | **HR (95% CI)** | **P-value** | **HR (95% CI)** | **P-value** |
| Rural-residing | 0.96 (0.93, 1.00) | 0.051 | 1.20 (1.08, 1.34) | <0.001 | 1.22 (1.05, 1.42) | 0.009 |
| Age ≥ 65 (years) | 1.01 (1.00, 1.01) | <0.001 | 1.03 (1.03, 1.04) | <0.001 | 1.03 (1.02, 1.04) | <0.001 |
| Male | 1.12 (1.02, 1.23) | 0.020 | 1.70 (1.17, 2.47) | 0.005 | 1.83 (1.05, 3.18) | 0.033 |
| **Race** |  |  |  |  |  |  |
| Black vs White | 0.93 (0.89, 0.97) | <0.001 | 0.75 (0.66, 0.86) | <0.001 | 0.74 (0.61, 0.89) | 0.001 |
| Other vs White | 0.94 (0.87, 1.00) | 0.066 | 0.88 (0.72, 1.07) | 0.197 | 0.91 (0.68, 1.21) | 0.524 |
| **BMI** |  |  |  |  |  |  |
| <21 vs 21-24.9 | 1.08 (1.02, 1.14) | 0.005 | 1.61 (1.40, 1.84) | <0.001 | 1.41 (1.16, 1.71) | <0.001 |
| 25-29.9 vs 21-24.9 | 0.88 (0.84, 0.92) | <0.001 | 0.90 (0.79, 1.02) | 0.100 | 0.96 (0.81, 1.15) | 0.682 |
| 30-34.9 vs 21-24.9 | 0.83 (0.79, 0.87) | <0.001 | 0.70 (0.60, 0.82) | <0.001 | 0.83 (0.67, 1.04) | 0.103 |
| 35+ vs 21-24.9 | 0.80 (0.76, 0.85) | <0.001 | 0.70 (0.58, 0.85) | <0.001 | 0.81 (0.63, 1.06) | 0.128 |
| Missing vs 21-24.9 | 0.58 (0.29, 1.18) | 0.133 | 2.46 (1.17, 5.16) | 0.017 | 2.63 (0.63, 11.06) | 0.186 |
| Medicaid | 1.05 (0.96, 1.15) | 0.259 | 0.90 (0.66, 1.22) | 0.501 | 1.07 (0.72, 1.61) | 0.729 |
| Medicare | 0.59 (0.57, 0.61) | <0.001 | 0.18 (0.16, 0.20) | <0.001 | 0.28 (0.24, 0.32) | <0.001 |
| **Lives Alone** |  |  |  |  |  |  |
| No vs Yes | 1.08 (1.03, 1.12) | <0.001 | 1.25 (1.10, 1.43) | <0.001 | 1.48 (1.22, 1.80) | <0.001 |
| Unknown vs Yes | 0.83 (0.77, 0.90) | <0.001 | 0.78 (0.60, 1.02) | 0.069 | 1.18 (0.82, 1.70) | 0.373 |
| **Marital Status** |  |  |  |  |  |  |
| Widowed vs. Married | 1.07 (1.01, 1.14) | 0.014 | 0.97 (0.83, 1.15) | 0.752 | 1.03 (0.83, 1.29) | 0.794 |
| Other vs. Married | 1.08 (1.04, 1.12) | <0.001 | 0.99 (0.88, 1.10) | 0.808 | 0.98 (0.84, 1.14) | 0.797 |
| Discharged from ICU | 1.06 (0.99, 1.14) | 0.098 | 1.21 (0.98, 1.50) | 0.076 | 0.99 (0.72, 1.35) | 0.927 |
| Dementia (ICD-10 in year prior) | 1.15 (1.09, 1.21) | <0.001 | 0.89 (0.77, 1.04) | 0.159 | 0.72 (0.57, 0.89) | 0.003 |
| Other Significant Endocrine & Metabolic Disorders | 1.10 (1.05, 1.15) | <0.001 | 1.16 (1.02, 1.32) | 0.023 | 1.20 (1.00, 1.45) | 0.055 |
| Psychiatric comorbidity | 1.16 (1.12, 1.20) | <0.001 | 0.96 (0.87, 1.07) | 0.470 | 0.88 (0.76, 1.01) | 0.067 |
| Cardiorespiratory Failure and Shock | 1.27 (1.21, 1.33) | <0.001 | 1.26 (1.10, 1.43) | <0.001 | 1.11 (0.93, 1.33) | 0.238 |
| Coronary atherosclerosis or angina, cerebrovascular disease | 1.18 (1.14, 1.23) | <0.001 | 1.05 (0.94, 1.17) | 0.420 | 0.93 (0.79, 1.08) | 0.344 |

**Supplemental Table 8. (continued)**

| **Characteristic** | **Readmission** | | **Mortality** | | **Mortality after Readmission** | |
| --- | --- | --- | --- | --- | --- | --- |
|  | **HR (95% CI)** | **P-value** | **HR (95% CI)** | **P-value** | **HR (95% CI)** | **P-value** |
| Arrhythmias or Conduction Disorders | 1.14 (1.10, 1.18) | <0.001 | 0.93 (0.84, 1.03) | 0.168 | 0.90 (0.78, 1.03) | 0.128 |
| Chronic Obstructive Pulmonary Disease | 1.10 (1.06, 1.14) | <0.001 | 1.11 (1.00, 1.23) | 0.060 | 1.01 (0.87, 1.17) | 0.918 |
| Fibrosis of Lunch and Other Chronic Lung Disorders | 1.11 (1.07, 1.15) | <0.001 | 1.04 (0.94, 1.16) | 0.446 | 0.96 (0.82, 1.11) | 0.553 |
| Dialysis Status | 1.41 (1.31, 1.53) | <0.001 | 1.48 (1.19, 1.84) | <0.001 | 1.22 (0.90, 1.63) | 0.195 |

**Supplemental Table 9.** Individual frailty models for each outcome censored at 90 days with Inverse-Probability of Treatment Weights (IPTW) using stabilized and truncated weights (99^th^ percentile).

| **Characteristic** | **Readmission** | | **Mortality** | | **Mortality after Readmission** | |
| --- | --- | --- | --- | --- | --- | --- |
|  | **HR (95% CI)** | **P-value** | **HR (95% CI)** | **P-value** | **HR (95% CI)** | **P-value** |
| Rural-residing | 0.96 (0.93, 0.98) | 0.002 | 1.12 (1.05, 1.19) | <0.001 | 1.18 (1.10, 1.27) | <0.001 |
| Age ≥ 65 (years) | 1.01 (1.00, 1.01) | <0.001 | 1.03 (1.03, 1.03) | <0.001 | 1.03 (1.02, 1.03) | <0.001 |
| Male | 1.10 (1.03, 1.18) | 0.006 | 1.46 (1.20, 1.77) | <0.001 | 1.42 (1.11, 1.81) | 0.005 |
| **Race** |  |  |  |  |  |  |
| Black vs White | 0.99 (0.96, 1.02) | 0.377 | 0.82 (0.76, 0.88) | <0.001 | 0.84 (0.77, 0.92) | <0.001 |
| Other vs White | 0.96 (0.91, 1.01) | 0.110 | 0.94 (0.84, 1.05) | 0.243 | 0.96 (0.83, 1.10) | 0.561 |
| **BMI** |  |  |  |  |  |  |
| <21 vs 21-24.9 | 1.08 (1.03, 1.12) | <0.001 | 1.47 (1.36, 1.58) | <0.001 | 1.40 (1.28, 1.54) | <0.001 |
| 25-29.9 vs 21-24.9 | 0.89 (0.86, 0.92) | <0.001 | 0.82 (0.76, 0.88) | <0.001 | 0.90 (0.83, 0.99) | 0.024 |
| 30-34.9 vs 21-24.9 | 0.85 (0.82, 0.88) | <0.001 | 0.69 (0.63, 0.75) | <0.001 | 0.79 (0.71, 0.87) | <0.001 |
| 35+ vs 21-24.9 | 0.82 (0.79, 0.86) | <0.001 | 0.59 (0.53, 0.66) | <0.001 | 0.68 (0.60, 0.78) | <0.001 |
| Missing vs 21-24.9 | 0.39 (0.21, 0.74) | 0.004 | 1.44 (0.84, 2.45) | 0.184 | 1.49 (0.49, 4.50) | 0.481 |
| Medicaid | 1.06 (0.99, 1.13) | 0.083 | 0.70 (0.58, 0.84) | <0.001 | 0.69 (0.54, 0.87) | 0.002 |
| Medicare | 0.57 (0.56, 0.59) | <0.001 | 0.18 (0.17, 0.19) | <0.001 | 0.27 (0.26, 0.29) | <0.001 |
| **Lives Alone** |  |  |  |  |  |  |
| No vs Yes | 1.05 (1.01, 1.08) | 0.005 | 1.10 (1.02, 1.18) | 0.012 | 1.10 (1.00, 1.20) | 0.045 |
| Unknown vs Yes | 0.81 (0.76, 0.85) | <0.001 | 0.75 (0.65, 0.87) | <0.001 | 0.92 (0.77, 1.10) | 0.361 |
| **Marital Status** |  |  |  |  |  |  |
| Widowed vs. Married | 1.05 (1.01, 1.10) | 0.014 | 0.89 (0.81, 0.98) | 0.013 | 0.87 (0.78, 0.98) | 0.020 |
| Other vs. Married | 1.06 (1.03, 1.09) | <0.001 | 0.96 (0.90, 1.02) | 0.160 | 0.99 (0.85, 1.16) | 0.922 |
| Discharged from ICU | 0.99 (0.93, 1.04) | 0.626 | 1.04 (0.92, 1.18) | 0.493 | 0.90 (0.81, 1.00) | 0.054 |
| Dementia (ICD-10 in year prior) | 1.14 (1.10, 1.19) | <0.001 | 1.04 (0.95, 1.13) | 0.385 | 1.07 (0.98, 1.18) | 0.127 |
| Other Significant Endocrine & Metabolic Disorders | 1.09 (1.06, 1.13) | <0.001 | 1.13 (1.05, 1.21) | <0.001 | 0.85 (0.79, 0.91) | <0.001 |
| Psychiatric comorbidity | 1.16 (1.13, 1.19) | <0.001 | 0.94 (0.89, 0.99) | 0.028 | 1.12 (1.02, 1.22) | 0.012 |
| Cardiorespiratory Failure and Shock | 1.31 (1.27, 1.36) | <0.001 | 1.28 (1.19, 1.38) | <0.001 | 0.90 (0.84, 0.98) | 0.011 |
| Coronary atherosclerosis or angina, cerebrovascular disease | 1.23 (1.20, 1.27) | <0.001 | 1.02 (0.96, 1.09) | 0.523 | 0.99 (0.85, 1.16) | 0.922 |

**Supplemental Table 9. (continued)**

| **Characteristic** | **Readmission** | | **Mortality** | | **Mortality after Readmission** | |
| --- | --- | --- | --- | --- | --- | --- |
|  | **HR (95% CI)** | **P-value** | **HR (95% CI)** | **P-value** | **HR (95% CI)** | **P-value** |
| Arrhythmias or Conduction Disorders | 1.18 (1.15, 1.21) | <0.001 | 1.04 (0.98, 1.10) | 0.215 | 0.97 (0.90, 1.04) | 0.351 |
| Chronic Obstructive Pulmonary Disease | 1.14 (1.11, 1.17) | <0.001 | 1.11 (1.04, 1.17) | <0.001 | 1.02 (0.95, 1.10) | 0.508 |
| Fibrosis of Lunch and Other Chronic Lung Disorders | 1.09 (1.06, 1.12) | <0.001 | 1.05 (0.99, 1.12) | 0.084 | 1.01 (0.94, 1.08) | 0.841 |
| Dialysis Status | 1.44 (1.36, 1.52) | <0.001 | 1.45 (1.28, 1.64) | <0.001 | 1.18 (1.02, 1.36) | 0.029 |

**Supplemental Table 10.** Individual frailty models for each outcome censored at 1 year with Inverse-Probability of Treatment Weights (IPTW) using stabilized and truncated weights (99^th^ percentile)

| **Characteristic** | **Readmission** | | **Mortality** | | **Mortality after Readmission** | |
| --- | --- | --- | --- | --- | --- | --- |
|  | **HR (95% CI)** | **P-value** | **HR (95% CI)** | **P-value** | **HR (95% CI)** | **P-value** |
| Rural-residing | 0.94 (0.92, 0.96) | <0.001 | 1.08 (1.04, 1.12) | <0.001 | 1.13 (1.08, 1.17) | <0.001 |
| Age ≥ 65 (years) | 1.01 (1.01, 1.01) | <0.001 | 1.04 (1.04, 1.04) | <0.001 | 1.03 (1.03, 1.04) | <0.001 |
| Male | 1.08 (1.03, 1.13) | 0.002 | 1.38 (1.24, 1.54) | <0.001 | 1.32 (1.16, 1.49) | <0.001 |
| **Race** |  |  |  |  |  |  |
| Black vs White | 1.02 (0.99, 1.04) | 0.144 | 0.88 (0.85, 0.92) | <0.001 | 0.88 (0.84, 0.92) | <0.001 |
| Other vs White | 0.95 (0.92, 0.99) | 0.016 | 0.91 (0.85, 0.97) | 0.006 | 0.94 (0.87, 1.02) | 0.125 |
| **BMI** |  |  |  |  |  |  |
| <21 vs 21-24.9 | 1.10 (1.07, 1.14) | <0.001 | 1.34 (1.28, 1.40) | <0.001 | 1.25 (1.18, 1.31) | <0.001 |
| 25-29.9 vs 21-24.9 | 0.90 (0.88, 0.92) | <0.001 | 0.83 (0.79, 0.86) | <0.001 | 0.87 (0.83, 0.91) | <0.001 |
| 30-34.9 vs 21-24.9 | 0.88 (0.86, 0.90) | <0.001 | 0.71 (0.68, 0.75) | <0.001 | 0.77 (0.72, 0.81) | <0.001 |
| 35+ vs 21-24.9 | 0.88 (0.85, 0.90) | <0.001 | 0.65 (0.61, 0.69) | <0.001 | 0.69 (0.64, 0.74) | <0.001 |
| Missing vs 21-24.9 | 0.33 (0.20, 0.54) | <0.001 | 0.79 (0.53, 1.19) | 0.262 | 0.82 (0.34, 2.01) | 0.671 |
| Medicaid | 1.06 (1.01, 1.11) | 0.015 | 0.65 (0.58, 0.72) | <0.001 | 0.62 (0.54, 0.70) | <0.001 |
| Medicare | 0.57 (0.56, 0.59) | <0.001 | 0.14 (0.13, 0.14) | <0.001 | 0.18 (0.18, 0.19) | <0.001 |
| **Lives Alone** |  |  |  |  |  |  |
| No vs Yes | 1.03 (1.01, 1.06) | 0.008 | 1.06 (1.02, 1.10) | 0.008 | 1.08 (1.03, 1.14) | 0.001 |
| Unknown vs Yes | 0.82 (0.78, 0.85) | <0.001 | 0.76 (0.70, 0.83) | <0.001 | 0.92 (0.83, 1.00) | 0.059 |
| **Marital Status** |  |  |  |  |  |  |
| Widowed vs. Married | 1.08 (1.04, 1.11) | <0.001 | 0.93 (0.89, 0.98) | 0.011 | 0.92 (0.87, 0.98) | 0.010 |
| Other vs. Married | 1.06 (1.04, 1.09) | <0.001 | 0.96 (0.93, 1.00) | 0.044 | 0.95 (0.92, 0.99) | 0.026 |
| Discharged from ICU | 0.98 (0.94, 1.02) | 0.234 | 0.98 (0.91, 1.06) | 0.587 | 0.96 (0.88, 1.05) | 0.399 |
| Dementia (ICD-10 in year prior) | 1.16 (1.13, 1.20) | <0.001 | 1.14 (1.09, 1.20) | <0.001 | 1.06 (1.00, 1.11) | 0.043 |
| Other Significant Endocrine & Metabolic Disorders | 1.09 (1.07, 1.12) | <0.001 | 1.11 (1.07, 1.16) | <0.001 | 1.08 (1.03, 1.13) | 0.002 |
| Psychiatric comorbidity | 1.18 (1.15, 1.20) | <0.001 | 1.00 (0.97, 1.03) | 0.998 | 0.92 (0.88, 0.95) | <0.001 |
| Cardiorespiratory Failure and Shock | 1.33 (1.29, 1.36) | <0.001 | 1.28 (1.23, 1.33) | <0.001 | 1.14 (1.09, 1.19) | <0.001 |
| Coronary atherosclerosis or angina, cerebrovascular disease | 1.27 (1.24, 1.29) | <0.001 | 1.15 (1.11, 1.19) | <0.001 | 1.03 (0.99, 1.08) | 0.146 |

**Supplemental Table 10. (continued)**

| **Characteristic** | **Readmission** | | **Mortality** | | **Mortality after Readmission** | |
| --- | --- | --- | --- | --- | --- | --- |
|  | **HR (95% CI)** | **P-value** | **HR (95% CI)** | **P-value** | **HR (95% CI)** | **P-value** |
| Arrhythmias or Conduction Disorders | 1.18 (1.16, 1.20) | <0.001 | 1.06 (1.03, 1.10) | <0.001 | 1.00 (0.97, 1.04) | 0.853 |
| Chronic Obstructive Pulmonary Disease | 1.18 (1.16, 1.20) | <0.001 | 1.18 (1.14, 1.22) | <0.001 | 1.09 (1.05, 1.14) | <0.001 |
| Fibrosis of Lunch and Other Chronic Lung Disorders | 1.10 (1.08, 1.12) | <0.001 | 1.07 (1.03, 1.10) | <0.001 | 1.03 (0.99, 1.07) | 0.173 |
| Dialysis Status | 1.55 (1.48, 1.62) | <0.001 | 1.54 (1.43, 1.65) | <0.001 | 1.28 (1.18, 1.38) | <0.001 |
| Other Model Information |  |  |  |  |  |  |

**Supplemental Table 11.** (Sensitivity analysis) Semi-competing risk model censored at 30 days excluding hospice (N =90049, removed 9508, also indicator for missing BMI removed as too few, so 69 placed in BMI normal category)

| **Characteristic** | **Readmission** | | **Mortality** | | **Mortality after Readmission** | |
| --- | --- | --- | --- | --- | --- | --- |
|  | **HR (95% CI)** | **P-value** | **HR (95% CI)** | **P-value** | **HR (95% CI)** | **P-value** |
| Rural-residing | 0.95 (0.90, 1.00) | 0.050 | 1.25 (1.02, 1.53) | 0.031 | 1.49 (1.15, 1.93) | 0.002 |
| Age ≥ 65 (years) | 1.01 (1.00, 1.01) | 0.016 | 1.03 (1.02, 1.04) | <0.001 | 1.02 (1.00, 1.04) | 0.024 |
| Male | 1.17 (1.02, 1.33) | 0.021 | 1.86 (0.95, 3.65) | 0.070 | 1.23 (0.57, 2.66) | 0.600 |
| **Race** |  |  |  |  |  |  |
| Black vs White | 0.90 (0.85, 0.95) | <0.001 | 0.92 (0.73, 1.16) | 0.457 | 0.63 (0.44, 0.89) | 0.009 |
| Other vs White | 0.91 (0.82, 1.00) | 0.058 | 1.00 (0.69, 1.45) | 0.994 | 0.88 (0.53, 1.46) | 0.614 |
| **BMI** |  |  |  |  |  |  |
| <21 vs 21-24.9 | 1.05 (0.97, 1.15) | 0.246 | 1.68 (1.28, 2.22) | <0.001 | 1.06 (0.71, 1.57) | 0.786 |
| 25-29.9 vs 21-24.9 | 0.85 (0.80, 0.91) | <0.001 | 0.90 (0.70, 1.16) | 0.411 | 0.83 (0.60, 1.14) | 0.240 |
| 30-34.9 vs 21-24.9 | 0.81 (0.76, 0.88) | <0.001 | 0.65 (0.47, 0.89) | 0.007 | 0.77 (0.53, 1.13) | 0.182 |
| 35+ vs 21-24.9 | 0.81 (0.74, 0.87) | <0.001 | 0.91 (0.65, 1.28) | 0.599 | 0.83 (0.54, 1.28) | 0.404 |
| Medicaid | 1.10 (0.97, 1.24) | 0.144 | 0.99 (0.58, 1.67) | 0.955 | 0.72 (0.32, 1.65) | 0.436 |
| Medicare | 0.59 (0.55, 0.63) | <0.001 | 0.12 (0.10, 0.15) | <0.001 | 0.22 (0.18, 0.29) | <0.001 |
| **Lives Alone** |  |  |  |  |  |  |
| No vs Yes | 1.04 (0.98, 1.11) | 0.188 | 0.87 (0.69, 1.09) | 0.209 | 1.50 (1.07, 2.11) | 0.020 |
| Unknown vs Yes | 0.78 (0.70, 0.87) | <0.001 | 0.62 (0.40, 0.97) | 0.038 | 1.06 (0.56, 2.00) | 0.855 |
| **Marital Status** |  |  |  |  |  |  |
| Widowed vs. Married | 1.08 (0.99, 1.18) | 0.077 | 1.23 (0.90, 1.68) | 0.201 | 1.57 (1.07, 2.31) | 0.023 |
| Other vs. Married | 1.11 (1.05, 1.17) | <0.001 | 1.22 (0.98, 1.51) | 0.069 | 1.14 (0.86, 1.51) | 0.380 |
| Discharged from ICU | 1.11 (1.00, 1.24) | 0.056 | 1.42 (0.97, 2.08) | 0.073 | 0.97 (0.56, 1.68) | 0.907 |
| Dementia (ICD-10 in year prior) | 1.25 (1.15, 1.36) | <0.001 | 1.29 (0.95, 1.75) | 0.102 | 0.75 (0.48, 1.16) | 0.195 |
| Other Significant Endocrine & Metabolic Disorders | 1.12 (1.05, 1.19) | <0.001 | 1.08 (0.85, 1.36) | 0.550 | 1.42 (0.97, 2.06) | 0.071 |
| Psychiatric comorbidity | 1.25 (1.19, 1.31) | <0.001 | 1.00 (0.82, 1.22) | 0.990 | 0.86 (0.67, 1.11) | 0.255 |
| Cardiorespiratory Failure and Shock | 1.31 (1.22, 1.40) | <0.001 | 1.50 (1.16, 1.95) | 0.002 | 1.37 (1.00, 1.89) | 0.050 |
| Coronary atherosclerosis or angina, cerebrovascular disease | 1.29 (1.22, 1.36) | <0.001 | 1.13 (0.91, 1.39) | 0.272 | 1.27 (0.94, 1.73) | 0.122 |
| Arrhythmias or Conduction Disorders | 1.21 (1.15, 1.27) | <0.001 | 0.91 (0.75, 1.11) | 0.367 | 0.84 (0.65, 1.09) | 0.195 |

**Supplemental Table 11. (continued)**

| **Characteristic** | **Readmission** | | **Mortality** | | **Mortality after Readmission** | |
| --- | --- | --- | --- | --- | --- | --- |
|  | **HR (95% CI)** | **P-value** | **HR (95% CI)** | **P-value** | **HR (95% CI)** | **P-value** |
| Chronic Obstructive Pulmonary Disease | 1.12 (1.06, 1.18) | <0.001 | 1.20 (0.97, 1.47) | 0.090 | 1.04 (0.79, 1.35) | 0.801 |
| Fibrosis of Lunch and Other Chronic Lung Disorders | 1.14 (1.08, 1.20) | <0.001 | 1.03 (0.84, 1.27) | 0.770 | 0.98 (0.75, 1.28) | 0.873 |
| Dialysis Status | 1.73 (1.52, 1.97) | <0.001 | 1.69 (1.08, 2.67) | 0.023 | 2.51 (1.62, 3.87) | <0.001 |
| Other Model Information |  |  |  |  |  |  |
| Variance of frailty (95% CI) | 3.80 (3.15, 4.57) |  |  |  |  |  |
| Number of parameters | 82 |  |  |  |  |  |
| Log-likelihood | -82386.57 |  |  |  |  |  |

Note: Wald test p-values reported for model parameters.

**Supplemental Table 12.** (Sensitivity analysis) Semi-competing risk model censored at 90 days excluding hospice (N =90049, removed 9508, also indicator for missing BMI removed as too few, so 69 placed in BMI normal category)

| **Characteristic** | **Readmission** | | **Mortality** | | **Mortality after Readmission** | |
| --- | --- | --- | --- | --- | --- | --- |
|  | **HR (95% CI)** | **P-value** | **HR (95% CI)** | **P-value** | **HR (95% CI)** | **P-value** |
| Rural-residing | 0.95 (0.92, 0.98) | 0.001 | 1.11 (0.98, 1.27) | 0.109 | 1.33 (1.19, 1.47) | <0.001 |
| Age ≥ 65 (years) | 1.00 (1.00, 1.01) | 0.001 | 1.03 (1.02, 1.04) | <0.001 | 1.03 (1.02, 1.04) | <0.001 |
| Male | 1.10 (1.02, 1.19) | 0.016 | 1.78 (1.15, 2.76) | 0.010 | 1.35 (0.96, 1.89) | 0.089 |
| **Race** |  |  |  |  |  |  |
| Black vs White | 0.98 (0.94, 1.01) | 0.187 | 0.93 (0.80, 1.08) | 0.345 | 0.77 (0.67, 0.88) | <0.001 |
| Other vs White | 0.94 (0.88, 1.00) | 0.044 | 1.02 (0.81, 1.29) | 0.880 | 0.88 (0.71, 1.08) | 0.212 |
| **BMI** |  |  |  |  |  |  |
| <21 vs 21-24.9 | 1.06 (1.01, 1.12) | 0.022 | 1.55 (1.30, 1.85) | <0.001 | 1.24 (1.07, 1.44) | 0.005 |
| 25-29.9 vs 21-24.9 | 0.89 (0.86, 0.93) | <0.001 | 0.81 (0.69, 0.95) | 0.010 | 0.83 (0.73, 0.94) | 0.004 |
| 30-34.9 vs 21-24.9 | 0.85 (0.82, 0.89) | <0.001 | 0.75 (0.62, 0.91) | 0.004 | 0.80 (0.69, 0.93) | 0.004 |
| 35+ vs 21-24.9 | 0.84 (0.80, 0.88) | <0.001 | 0.79 (0.63, 0.98) | 0.033 | 0.74 (0.62, 0.89) | 0.001 |
| Medicaid | 1.10 (1.02, 1.18) | 0.016 | 0.80 (0.55, 1.15) | 0.228 | 0.60 (0.42, 0.85) | 0.005 |
| Medicare | 0.57 (0.55, 0.59) | <0.001 | 0.13 (0.11, 0.14) | <0.001 | 0.21 (0.19, 0.24) | <0.001 |
| **Lives Alone** |  |  |  |  |  |  |
| No vs Yes | 1.03 (0.99, 1.07) | 0.189 | 0.90 (0.78, 1.05) | 0.170 | 1.06 (0.93, 1.20) | 0.395 |
| Unknown vs Yes | 0.78 (0.73, 0.83) | <0.001 | 0.66 (0.49, 0.88) | 0.005 | 0.88 (0.69, 1.13) | 0.309 |
| **Marital Status** |  |  |  |  |  |  |
| Widowed vs. Married | 1.07 (1.02, 1.13) | 0.009 | 1.17 (1.02, 1.34) | 0.023 | 0.92 (0.77, 1.08) | 0.309 |
| Other vs. Married | 1.08 (1.04, 1.12) | <0.001 | 1.22 (0.98, 1.51) | 0.069 | 0.88 (0.79, 0.99) | 0.028 |
| Discharged from ICU | 0.99 (0.93, 1.06) | 0.871 | 1.15 (0.89, 1.50) | 0.293 | 0.98 (0.79, 1.23) | 0.884 |
| Dementia (ICD-10 in year prior) | 1.19 (1.13, 1.25) | <0.001 | 1.24 (1.02, 1.50) | 0.029 | 0.92 (0.78, 1.07) | 0.279 |
| Other Significant Endocrine & Metabolic Disorders | 1.11 (1.07, 1.16) | <0.001 | 1.05 (0.90, 1.22) | 0.535 | 1.05 (0.91, 1.20) | 0.511 |
| Psychiatric comorbidity | 1.21 (1.18, 1.25) | <0.001 | 0.95 (0.83, 1.07) | 0.394 | 0.84 (0.76, 0.93) | 0.001 |
| Cardiorespiratory Failure and Shock | 1.34 (1.29, 1.40) | <0.001 | 1.52 (1.28, 1.79) | <0.001 | 1.33 (1.17, 1.51) | <0.001 |
| Coronary atherosclerosis or angina, cerebrovascular disease | 1.29 (1.25, 1.34) | <0.001 | 1.04 (0.91, 1.19) | 0.565 | 1.02 (0.91, 1.15) | 0.759 |
| Arrhythmias or Conduction Disorders | 1.23 (1.19, 1.27) | <0.001 | 1.08 (0.95, 1.23) | 0.215 | 1.03 (0.93, 1.15) | 0.531 |
| Chronic Obstructive Pulmonary Disease | 1.16 (1.12, 1.20) | <0.001 | 1.15 (1.01, 1.31) | 0.041 | 1.05 (0.94, 1.17) | 0.404 |
| Fibrosis of Lunch and Other Chronic Lung Disorders | 1.10 (1.06, 1.13) | <0.001 | 0.92 (0.80, 1.05) | 0.203 | 1.01 (0.91, 1.12) | 0.853 |
| Dialysis Status | 1.59 (1.48, 1.72) | <0.001 | 1.68 (1.27, 2.23) | <0.001 | 1.57 (1.29, 1.93) | <0.001 |
| Other Model Information |  |  |  |  |  |  |
| Variance of frailty (95% CI) | 0.69 (0.54, 0.87) |  |  |  |  |  |
| Number of parameters | 82 |  |  |  |  |  |
| Log-likelihood | -180405.2 |  |  |  |  |  |

Note: Wald test p-values reported for model parameters.

**Supplemental Table 13**. (Sensitivity analysis) Semi-competing risk model censored at 1 year excluding hospice (N =90049, removed 9508, also indicator for missing BMI removed as too few, so 69 placed in BMI normal category)

| **Characteristic** | **Readmission** | | **Mortality** | | **Mortality after Readmission** | |
| --- | --- | --- | --- | --- | --- | --- |
|  | **HR (95% CI)** | **P-value** | **HR (95% CI)** | **P-value** | **HR (95% CI)** | **P-value** |
| Rural-residing | 0.93 (0.91, 0.95) | <0.001 | 1.06 (0.97, 1.15) | 0.209 | 1.17 (1.12, 1.23) | <0.001 |
| Age ≥ 65 (years) | 1.01 (1.01, 1.01) | <0.001 | 1.04 (1.04, 1.05) | <0.001 | 1.04 (1.04, 1.04) | <0.001 |
| Male | 1.08 (1.02, 1.14) | 0.005 | 1.66 (1.26, 2.19) | <0.001 | 1.30 (1.12, 1.51) | 0.001 |
| **Race** |  |  |  |  |  |  |
| Black vs White | 1.03 (1.00, 1.05) | 0.040 | 0.95 (0.87, 1.05) | 0.316 | 0.84 (0.79, 0.89) | <0.001 |
| Other vs White | 0.94 (0.90, 0.98) | 0.004 | 0.89 (0.76, 1.04) | 0.139 | 0.89 (0.81, 0.97) | 0.011 |
| **BMI** |  |  |  |  |  |  |
| <21 vs 21-24.9 | 1.11 (1.07, 1.15) | <0.001 | 1.46 (1.30, 1.64) | <0.001 | 1.24 (1.16, 1.33) | <0.001 |
| 25-29.9 vs 21-24.9 | 0.90 (0.88, 0.93) | <0.001 | 0.85 (0.77, 0.94) | 0.001 | 0.87 (0.82, 0.92) | <0.001 |
| 30-34.9 vs 21-24.9 | 0.90 (0.87, 0.92) | <0.001 | 0.76 (0.67, 0.86) | <0.001 | 0.79 (0.73, 0.84) | <0.001 |
| 35+ vs 21-24.9 | 0.90 (0.87, 0.93) | <0.001 | 0.84 (0.73, 0.96) | 0.014 | 0.74 (0.68, 0.80) | <0.001 |
| Medicaid | 1.08 (1.03, 1.14) | 0.002 | 0.72 (0.56, 0.93) | 0.010 | 0.56 (0.48, 0.66) | <0.001 |
| Medicare | 0.57 (0.56, 0.59) | <0.001 | 0.10 (0.09, 0.11) | <0.001 | 0.15 (0.14, 0.16) | <0.001 |
| **Lives Alone** |  |  |  |  |  |  |
| No vs Yes | 1.02 (0.99, 1.05) | 0.145 | 0.87 (0.80, 0.96) | 0.006 | 1.07 (1.01, 1.14) | 0.021 |
| Unknown vs Yes | 0.81 (0.77, 0.85) | <0.001 | 0.61 (0.50, 0.73) | <0.001 | 0.91 (0.81, 1.01) | 0.077 |
| **Marital Status** |  |  |  |  |  |  |
| Widowed vs. Married | 1.10 (1.06, 1.14) | <0.001 | 0.95 (0.83, 1.08) | 0.445 | 0.94 (0.87, 1.01) | 0.097 |
| Other vs. Married | 1.07 (1.05, 1.10) | <0.001 | 1.01 (0.92, 1.10) | 0.902 | 0.96 (0.91, 1.01) | 0.091 |
| Discharged from ICU | 0.97 (0.93, 1.02) | 0.229 | 1.06 (0.89, 1.26) | 0.542 | 0.97 (0.87, 1.07) | 0.533 |
| Dementia (ICD-10 in year prior) | 1.20 (1.16, 1.25) | <0.001 | 1.29 (1.14, 1.46) | <0.001 | 1.12 (1.05, 1.20) | 0.001 |
| Other Significant Endocrine & Metabolic Disorders | 1.11 (1.08, 1.14) | <0.001 | 0.95 (0.87, 1.05) | 0.307 | 1.07 (1.01, 1.14) | 0.032 |
| Psychiatric comorbidity | 1.20 (1.18, 1.23) | <0.001 | 1.03 (0.95, 1.12) | 0.458 | 0.91 (0.87, 0.96) | <0.001 |
| Cardiorespiratory Failure and Shock | 1.34 (1.30, 1.38) | <0.001 | 1.43 (1.27, 1.60) | <0.001 | 1.25 (1.18, 1.33) | <0.001 |
| Coronary atherosclerosis or angina, cerebrovascular disease | 1.32 (1.29, 1.35) | <0.001 | 1.14 (1.04, 1.24) | 0.003 | 1.08 (1.02, 1.14) | 0.005 |
| Arrhythmias or Conduction Disorders | 1.21 (1.18, 1.23) | <0.001 | 1.01 (0.93, 1.10) | 0.780 | 1.06 (1.02, 1.12) | 0.009 |

**Supplemental Table 13**. (Sensitivity analysis) Semi-competing risk model censored at 1 year excluding hospice (N =90049, removed 9508 with any hospice), also indicator for missing BMI removed as too few (so 69 placed in BMI normal category)

| **Characteristic** | **Readmission** | | **Mortality** | | **Mortality after Readmission** | |
| --- | --- | --- | --- | --- | --- | --- |
|  | **HR (95% CI)** | **P-value** | **HR (95% CI)** | **P-value** | **HR (95% CI)** | **P-value** |
| Chronic Obstructive Pulmonary Disease | 1.20 (1.17, 1.23) | <0.001 | 1.19 (1.09, 1.30) | <0.001 | 1.11 (1.06, 1.17) | <0.001 |
| Fibrosis of Lunch and Other Chronic Lung Disorders | 1.10 (1.08, 1.12) | <0.001 | 0.92 (0.84, 1.01) | 0.067 | 1.04 (0.99, 1.09) | 0.127 |
| Dialysis Status | 1.67 (1.59, 1.76) | <0.001 | 1.69 (1.39, 2.07) | <0.001 | 1.51 (1.38, 1.66) | <0.001 |
| Other Model Information |  |  |  |  |  |  |
| Variance of frailty (95% CI) | 0.17 (0.13, 0.22) |  |  |  |  |  |
| Number of parameters | 82 |  |  |  |  |  |
| Log-likelihood | -411915.9 |  |  |  |  |  |

Note: Wald test p-values reported for model parameters.

**Supplemental Table 14**. Major diagnostic categories for Index Admission

| **Characteristic** | **Rural/Highly Rural** | **Urban** | **Standardized**  **Difference** |
| --- | --- | --- | --- |
| Diseases of the blood and blood forming organs and certain disorders involving the immune mechanism | 546 (1.9) | 1397 (2.0) | -0.2 |
| Diseases of the circulatory system | 8331 (29.5) | 20552 (28.8) | 1.5 |
| Diseases of the digestive system | 2783 (9.9) | 6910 (9.7) | 0.6 |
| Diseases of the ear and mastoid process | 94 (0.3) | 254 (0.4) | -0.4 |
| Endocrine, nutritional and metabolic diseases | 1338 (4.7) | 3549 (5.0) | -1.1 |
| Diseases of the eye and adnexa | 120 (0.4) | 324 (0.4) | -0.4 |
| Factors influencing health status and contact with health services | 50 (0.2) | 106 (0.2) | 0.7 |
| Diseases of the genitourinary system | 1858 (6.6) | 5029 (7.0) | -1.9 |
| Certain infectious and parasitic diseases | 2495 (8.8) | 6486 (9.1) | -0.9 |
| Injury, poisoning, and certain other consequences of external causes | 1108 (3.9) | 3000 (4.2) | -1.4 |
| Congenital malformations, deformations and chromosomal abnormalities | 10 (0.04) | 27 (0.04) | -0.1 |
| Mental, behavioral and neurodevelopmental disorders | 359 (1.3) | 1125 (1.6) | -2.6 |
| Diseases of the musculoskeletal system and connective tissue | 786 (2.8) | 2085 (2.9) | -0.8 |
| Neoplasms | 827 (2.9) | 2234 (3.1) | -1.2 |
| Diseases of the nervous system | 916 (3.3) | 2581 (3.6) | -2.1 |
| Diseases of the respiratory system | 4019 (14.2) | 8804 (12.3) | 5.6 |
| Diseases of the skin and subcutaneous tissue | 872 (3.1) | 1895 (2.7) | 2.6 |
| Symptoms, signs and abnormal clinical and laboratory findings, NEC | 1526 (5.4) | 4447 (6.2) | -3.5 |
| Not Classified | 189 (0.7) | 525 (0.7) | -0.8 |

**Supplemental Table 15**. Semi-competing risk model censored at 30 days and logistic regression for readmission at 30 days for comparison only

| **Characteristic** | **Readmission (Semi-competing risk)** | | **Readmission (Logistic)**  **AUC = 0.619** | |
| --- | --- | --- | --- | --- |
|  | **HR (95% CI)** | **P-value** | **OR (95% CI)** | **P-value** |
| Rural-residing | 0.95 (0.91, 1.00) | 0.045 | 0.96 (0.92, 1.00) | 0.033 |
| Age ≥ 65 (years) | 1.01 (1.01, 1.01) | <0.001 | 1.01 (1.00, 1.01) | <0.001 |
| Male | 1.17 (1.04, 1.31) | 0.009 | 1.13 (1.03, 1.25) | 0.014 |
| Race |  |  |  |  |
| Black vs White | 0.88 (0.83, 0.92) | <0.001 | 0.91 (0.87, 0.95) | <0.001 |
| Other vs White | 0.90 (0.82, 0.98) | 0.016 | 0.92 (0.85, 0.99) | 0.025 |
| BMI |  |  |  |  |
| <21 vs 21-24.9 | 1.13 (1.05, 1.21) | 0.001 | 1.10 (1.04, 1.17) | 0.002 |
| 25-29.9 vs 21-24.9 | 0.84 (0.79, 0.88) | <0.001 | 0.87 (0.83, 0.91) | <0.001 |
| 30-34.9 vs 21-24.9 | 0.77 (0.72, 0.82) | <0.001 | 0.82 (0.77, 0.86) | <0.001 |
| 35+ vs 21-24.9 | 0.75 (0.70, 0.81) | <0.001 | 0.79 (0.74, 0.84) | <0.001 |
| Missing vs 21-24.9 | 0.52 (0.22, 1.25) | 0.144 | 0.48 (0.22, 1.06) | 0.070 |
| Medicaid | 1.06 (0.95, 1.18) | 0.330 | 1.04 (0.94, 1.14) | 0.455 |
| Medicare | 0.49 (0.47, 0.52) | <0.001 | 0.56 (0.54, 0.58) | <0.001 |
| Lives Alone |  |  |  |  |
| No vs Yes | 1.09 (1.03, 1.15) | 0.002 | 1.07 (1.03, 1.13) | 0.002 |
| Unknown vs Yes | 0.79 (0.72, 0.87) | <0.001 | 0.82 (0.75, 0.89) | <0.001 |
| Marital Status |  |  |  |  |
| Widowed vs. Married | 1.10 (1.02, 1.18) | 0.014 | 1.08 (1.02, 1.15) | 0.014 |
| Other vs. Married | 1.10 (1.05, 1.16) | <0.001 | 1.09 (1.04, 1.13) | <0.001 |
| Discharged from ICU | 1.09 (1.00, 1.20) | 0.063 | 1.05 (0.97, 1.14) | 0.248 |
| Dementia (ICD-10 in year prior) | 1.26 (1.17, 1.35) | <0.001 | 1.17 (1.11, 1.24) | <0.001 |
| Other Significant Endocrine & Metabolic Disorders | 1.10 (1.04, 1.17) | 0.001 | 1.09 (1.04, 1.15) | <0.001 |
| Psychiatric comorbidity | 1.21 (1.16, 1.26) | <0.001 | 1.18 (1.14, 1.23) | <0.001 |
| Cardiorespiratory Failure and Shock | 1.37 (1.29, 1.46) | <0.001 | 1.31 (1.25, 1.37) | <0.001 |
| Coronary atherosclerosis or angina, cerebrovascular disease | 1.24 (1.18, 1.30) | <0.001 | 1.20 (1.15, 1.25) | <0.001 |

**Supplemental Table 15. (continued)**

| **Characteristic** | **Readmission (Semi-competing risk)** | | **Readmission (Logistic)**  **AUC = 0.619** | |
| --- | --- | --- | --- | --- |
|  | **HR (95% CI)** | **P-value** | **OR (95% CI)** | **P-value** |
| Arrhythmias or Conduction Disorders | 1.19 (1.13, 1.24) | <0.001 | 1.17 (1.12, 1.21) | <0.001 |
| Chronic Obstructive Pulmonary Disease | 1.12 (1.07, 1.17) | <0.001 | 1.11 (1.07, 1.15) | <0.001 |
| Fibrosis of Lunch and Other Chronic Lung Disorders | 1.14 (1.09, 1.19) | <0.001 | 1.13 (1.08, 1.17) | <0.001 |
| Dialysis Status | 1.65 (1.48, 1.84) | <0.001 | 1.50 (1.37, 1.64) | <0.001 |

**Supplemental Table 16.** Frequencies and Percents for Outcomes

| **Characteristic** | **Rural/Highly Rural**  **(n=28227)** | **Urban**  **(n=71330)** |
| --- | --- | --- |
| **Outcomes** |  |  |
| Readmission 30 days | 4360 (15.4) | 11280 (15.8) |
| Readmission 90 days | 8032 (28.5) | 21042 (29.5) |
| Readmission 365 days | 14532 (51.5) | 38529 (54.0) |
| Death 30 days | 574 (2.0) | 1173 (1.6) |
| Death 90 days | 1749 (6.2) | 3860 (5.4) |
| Death 365 days | 4971 (17.6) | 11768 (16.5) |
